# Metal–Metal Oxide Interaction Modulated Photocatalytic Methane Conversion

**DOI:** 10.1021/jacs.6c01783

**Published:** 2026-04-16

**Authors:** Yanzhao Zhang, Jiakang You, Kai Wang, Dazhi Yao, Haijiao Lu, Jitraporn Vongsvivut, Jingwei Hou, Peng Chen, Yonggang Jin, Gang Liu, Zhiliang Wang, Lianzhou Wang

**Affiliations:** † Nanomaterials Centre, School of Chemical Engineering and Australian Institute for Bioengineering and Nanotechnology, 1974 The University of Queensland, St Lucia, QLD 4072, Australia; ‡ Department of Applied Biology and Chemical Technology, The Hong Kong Polytechnic University, Kowloon 999077, Hong Kong SAR, China; § Commonwealth Scientific and Industrial Research Organization (CSIRO) Mineral Resources, 1 Technology Court, Pullenvale, QLD 4069, Australia; ∥ Infrared Microspectroscopy (IRM) Beamline, ANSTO-Australian Synchrotron, Clayton, VIC 3168, Australia; ⊥ Shenyang National Laboratory for Materials Science, Institute of Metal Research, Chinese Academy of Sciences, 72 Wenhua Road, Shenyan 110016, China; # School of Materials Science and Engineering, University of Science and Technology of China, 72 Wenhua Road, Shenyang 110016, China

## Abstract

Metal–metal oxide (M–MO) interactions are important in catalysis. However, insights into how such interactions modulate lattice oxygen activity and stabilize critical reaction intermediates are scarce. In this work, using photocatalytic oxidative coupling of methane (POCM) as an example, we develop a simple and predictive model that defines M–MO interactions using two key factors: oxygen vacancy formation energy (*E*_OV_) and the methyl (*CH_3_) adsorption energy difference (ΔE_*CH_3__) across metal and oxide sites. Interfacial coupling comodulates *E*_OV_ and ΔE_*CH_3__. *E*_OV_ governs lattice-oxygen reactivity and the initial C–H activation, while ΔE_*CH_3__ controls CH_3_ distribution between metal and oxide sites and thereby C–C coupling selectivity. Correlating *E*_OV_ and ΔE_*CH_3__ with activity and selectivity reveals a unifying principle. Efficient methane conversion requires moderately labile lattice oxygen whereas selective C–C bond formation demands a large ΔE_*CH_3__ to drive methyl coupling for multicarbon products. Specifically, a AgPd/TiO_2_ catalyst achieves an optimal balance in experimental testing, delivering over a methane conversion yield of 30 mmol g^–1^ h^–1^, a selectivity of 92% for C_2_ products, and an operation stability of around 160 h. More broadly, the *E*_OV_–ΔE_*CH_3__ framework provides a predictive descriptor map for M–MO photocatalysts selection in POCM. This study fills a critical gap by establishing a quantitative framework for M–MO interactions, identifying interfacial synergy as the principal determinant of performance, and enabling rational M–MO catalyst design.

## Graphical abstract

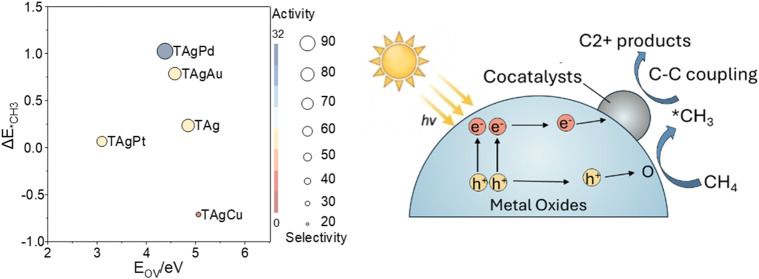

## Introduction

Metal–metal oxide (M–MO) interactions represent a fundamental design principle in functional materials, as interfacial charge transfer and electronic modulation at these junctions create properties that govern overall performance across diverse applications. Supported metals, exemplify this concept and are widely applied in heterogeneous catalysis,^1–4^ energy storage,^5^ gas sensing,^6^ and electronic devices.^7^ Their activity often arises from the unique synergy at the M–MO interface, where interactions are inherently bidirectional.^8–10^ On one hand, MO supports can modulate the electronic structure, dispersion, and stability of the metal component.^11,12^ On the other hand, deposited metals can profoundly change the MO substrate by inducing oxygen vacancies, modifying the band structure, and reshaping surface chemistry.^2,13,14^ The significance of M–MO interactions has received intensive attention in conventional catalysis, but remains insufficiently explored in the emerging photocatalysis process. Taking photocatalytic oxidative coupling of methane (POCM) as an example, a range of metallic catalysts, including single atoms,^10,15,16^ nanoparticles,^6,13^ nanoalloys,^2,9^ and compounds,^17,18^ have been employed to enhance photocatalytic performance. However, fundamental questions remain unclear on how M–MO interactions regulate the generation, stabilization, and conversion of key intermediates, which ultimately determine the activity and selectivity of POCM.

In POCM, lattice oxygen of MO is regarded as the active species for the initial cleavage of the C–H bond for the generation of key intermediates of methyl radicals (*CH_3_).^9,19^ The subsequent behavior of *CH_3_ is strongly influenced by the M–MO interaction. When *CH_3_ migrates to metal sites, C–C coupling can be promoted with the formation of C_2_ hydrocarbons such as ethane and ethylene (C_2_ generation pathway).^9,20–22^ If, however, *CH_3_ remains confined to the MO surface, it undergoes deep oxidation, producing CO_2_ as the major products (CO_2_ generation pathway). The reaction pathways are governed by the composition of metal and the MO substrate, as well as by the interactions between them. Previous research indicates that despite the same photocatalyst (e.g., TiO_2_), the methane conversion and product selectivity show a remarkable dependence on metal cocatalysts such as Au,^6,23^ Ag,^24^ Pt,^2,13^ and Pd.^25^ It is speculated that metal can dramatically influence metal oxides and regulate the reactivity of lattice oxygen. In turn, metal oxide can modulate the electronic structure of the metal cocatalyst, leading to tunable adsorption strength of surface intermediates. This has been reflected in product selectivity dependence on various metal oxides with Au as the cocatalysts.^10,21^ Despite the trial-and-error efforts, there is very limited understanding about the intrinsic features of M–MO interaction in a M–MO photocatalysts in determining the activity and selectivity in POCM.

On the other hand, the effects of cocatalysts can be strongly modulated by their composition and structure. For example, an AgAuCu alloy enhances ethylene production compared with monometallic Cu,^26^ because the incorporation of Ag and Au suppresses the formation of intermediates such as *C_2_H_5_ and *C_2_H_2_, thereby improving ethylene selectivity. Furthermore, the introduction of heterometallic sites induces electronic perturbations and reshapes the local chemical environment, which can facilitate the C–C coupling.^27^ The presence of multiple metal sites significantly modifies the adsorption energies of key intermediates, and therefore steers the reaction pathway. For instance, AuPd nanoalloys can stabilize *CH_2_ intermediates and thereby strongly promote ethylene formation.^28^

In this work, we move beyond the qualitative view of M–MO interaction as “strong or weak” and parametrize it with two transferable descriptors: oxygen vacancy formation energy (*E*_OV_) in MO and *CH_3_ adsorption energy difference (ΔE_*CH_3__) at the M–MO interface. Considering the high performance and modulational flexibility of sliver in reported references,^23,24^ density functional theory (DFT) calculations across a series of Ag-based metals supported on TiO_2_ shows a significant change of *E*_OV_ and ΔE_*CH_3__ depending on the bimetal compositions (Ag incorporated with Pd, Pt, Cu, and Au, denoted as TAgX, T represent TiO_2_, X indicates the same atomic amount of second metal composition). Further experimental evaluation on the POCM performance of the corresponding photocatalysts revealed strong relationships of activity *E*_OV_ and selectivity ΔE_*CH_3__, respectively. Specifically, small *E*_OV_ can promote methane activation, while large ΔE_*CH_3__ is beneficial for C_2_ products generation. Among them, TAgPd exhibited the most favorable interfacial properties, achieving optimized *E*_OV_ and ΔE_*CH_3__ values that translated into a methane conversion rate exceeding 30 mmol g^–1^ h^–1^ with 92% selectivity toward C_2_ hydrocarbons. Collectively, the long-standing activity-selectivity trade-off in POCM is quantitatively encoded by the *E*_OV_–ΔE_*CH_3__ relationship and programmable through M–MO interaction. This will provide a generic design principle of catalysts by transforming phenomenological study to a testable, predictive, and quantitative approach.

## Results and Discussion

### Theoretical Insights of Activity and Selectivity in POCM

In both C_2_ and CO_2_ generation pathways, the initial step involves cleavage of the first C–H bond in methane facilitated by lattice oxygen species of anatase, producing methyl radicals (*CH_3_) and surface hydrogen (*H) as shown in Figure 1a. The removal of *H species consumes lattice oxygen, generating water and leaving an oxygen vacancy.^19^ Thus, methane activation is intrinsically coupled to lattice oxygen consumption, making the *E*_OV_ a potential key parameter for evaluating methane conversion activity. Meanwhile, the *CH_3_ will be either absorbed on the MO surface or migrate to metal surface. For those *CH_3_ confined on the MO surface, are more likely to undergo further dehydrogenation and oxidation, eventually contributing to CO_2_ formation. In contrast, the migration of *CH_3_ to the metal cocatalyst surface increases the likelihood of C–C coupling for C_2_ products. We note, however, that metal-bonded *CH_3_ does not necessarily undergo coupling exclusively, as further dehydrogenation or other side reactions may also occur. Therefore, the key role of the cocatalyst is not to guarantee C_2_ formation from every adsorbed *CH_3_ species, but to provide a more coupling-favorable environment comparing to the oxidation-prone MO surface.^19^ From this perspective, the selectivity toward C_2_ products is expected to depend on the relative thermodynamic preference of methyl species for the metal surface (E_*CH_3_‑M_) versus the MO surface (E_*CH_3_‑MO_), which is quantified by the methyl adsorption energy difference (ΔE_*CH_3__ , = E_*CH_3_‑M_ – E_*CH_3_‑MO_). Accordingly, ΔE_*CH_3__ should be viewed as a descriptor of methyl partitioning between coupling-favorable and oxidation-prone sites, rather than as a complete descriptor of all possible downstream reactions of *CH_3_. Based on this understanding, we validate the proposed relationships through theoretical calculations and experimental verification.

**Figure 1:**
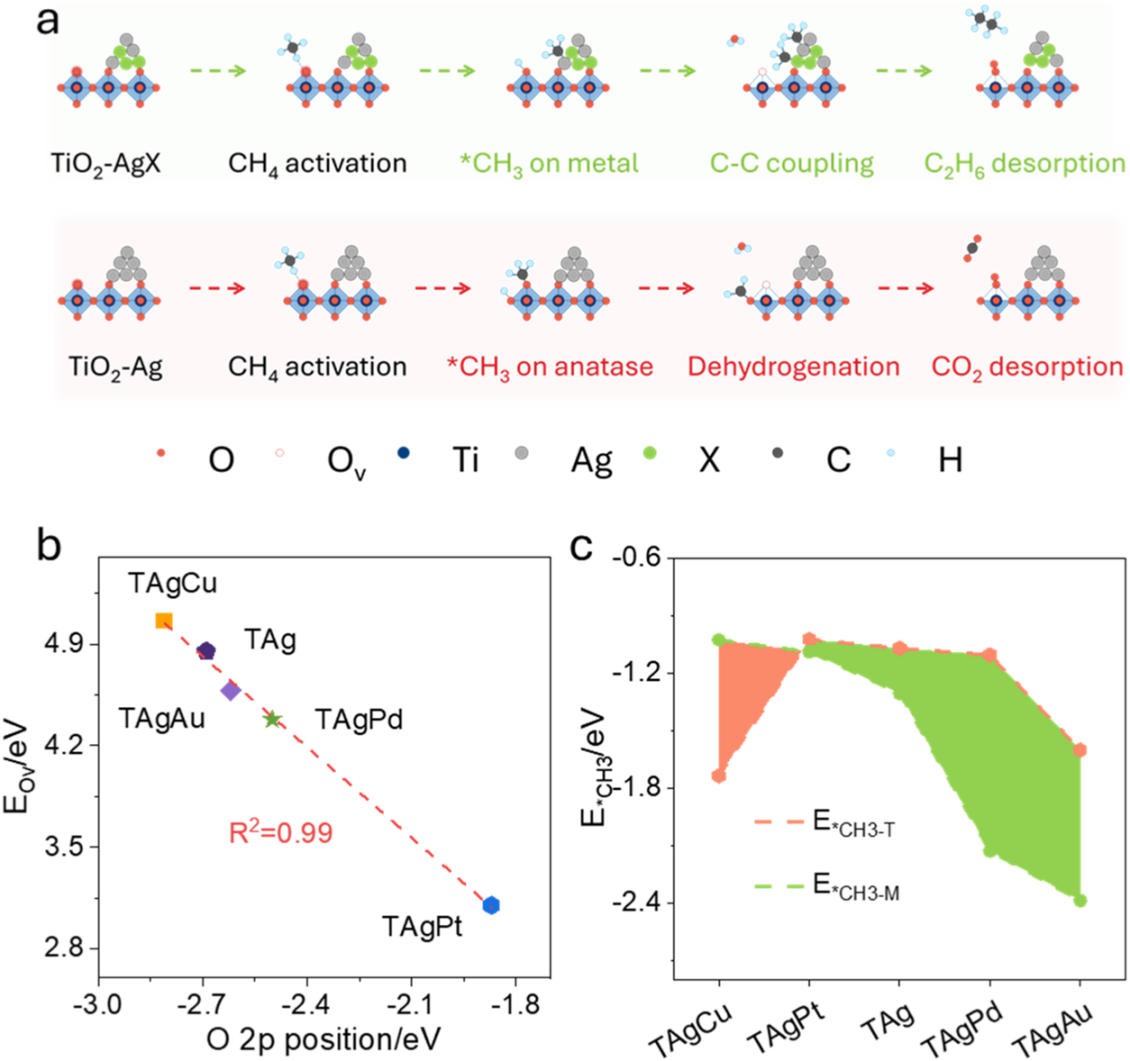
Proposed reaction mechanism and theoretical prediction of activity–selectivity relationships in POCM. (a) Schematic illustration of M–MO interaction between supported metal cocatalysts and the TiO_2_ lattice oxygen mechanism for POCM toward different products; (b) correlation between *E*_OV_ and the O 2p band onset, suggesting that O 2p band alignment governs oxygen vacancy formation; (c) adsorption energies of *CH_3_ on anatase TiO_2_ and different supported metals, where stronger *CH_3_ stabilization on AgPd is linked to enhanced surface coupling. The red and green areas indicate regions where methyl species exhibit stronger adsorption on anatase TiO_2_ and the supported metal sites, respectively.

To conduct the theoretical research, we build the different cocatalyst structures based on the anatase TiO_2_, shown in Figure S1 (Supporting Information, SI). The interaction between supported metals and the TiO_2_ lattice was found to significantly influence the O 2p band onset (the onset energy of the occupied O 2p states in the PDOS, referenced to the Fermi level), which in turn governs *E*_OV_. Since the valence band maximum (VBM) of TiO_2_ is dominated by O 2p states (Figure S2), an upward shift of the O 2p level can strengthen O 2p–Ti 3d antibonding hybridization, leading to weak Ti–O bonds with labile lattice oxygen for oxygen vacancy formation.^19^ Calculations reveal that Ag-loaded TiO_2_ exhibits an O 2p level at −2.69 eV, which is further shifted upward with Au (−2.62 eV), Pd (−2.50 eV), and Pt (−1.87 eV), or slightly downward with Cu (−2.81 eV). Such trend in O 2p level is consistent with their calculated *E*_OV_. By correlating the O 2p energy level and *E*_OV_ (Figure 1b), a good linear relationship is found where higher O 2p level leads to lower *E*_OV_. Based on this descriptor, TAgPt is predicted to deliver the highest lattice oxygen activity and may show the highest performance in methane conversion. Additionally, the calculated *E*_OV_ values are site-dependent: oxygen atoms far from the M–MO interface behaves similarly to those in pristine TiO_2_, while interfacial oxygen atoms are more easily removed. This result supports the viewpoint that the cocatalyst primarily perturbs and activates lattice oxygen in the interfacial region (Figure S3).

While *E*_OV_ is closely linked to methane activation and overall activity, product selectivity is dictated by the behavior of the *CH_3_ intermediates. For example, as the predominant C_2_ product, ethane (C_2_H_6_) is formed via coupling of two *CH_3_ species, whereas CO_2_ arises from their sequential oxidation (Figure 1a). The fate of *CH_3_ depends on its preferential adsorption: migration to metal sites favors C–C coupling, whereas retention on oxide surfaces leads to overoxidation. To capture this effect, we computed the *CH_3_ adsorption energy (E_*CH_3__) on both TiO_2_ (E_*CH_3_‑T_) and the supported metal surfaces (E_*CH_3_‑M_). As shown in Figure 1c, the binding strength on metal surfaces follows the order: TAgAu (−2.39 eV) > TAgPd (−2.13 eV) > TAg (−1.30 eV) > TAgPt (−1.09 eV) > TAgCu (−1.03 eV). In parallel, the adsorption strength on TiO_2_ was also significantly modulated by the supported metals, confirming strong electronic coupling between the oxide and metal components, see the different *CH_3_ adsorption structures in Figure S4. Generally, *CH_3_ prefers the site with stronger adsorption. For most systems (TAg, TAgAu, TAgPd, TAgPt), *CH_3_ adsorption is favored on the metal sites, while in TAgCu, *CH_3_ binds more strongly to TiO_2_. The proposed relative adsorption strength difference provides a rational descriptor for product selectivity. In systems where *CH_3_ preferentially adsorbs on metal sites, C–C coupling is favored, enhancing C_2_ selectivity. In contrast, when *CH_3_ remains confined to TiO_2_, deep oxidation dominates, lowering C_2_ yields. Based on this descriptor, TAgPd is predicted to exhibit the highest selectivity toward C_2_ products, whereas TAgCu is expected to show the lowest.

### Experimental Validation of Structure-Performance Relationships

To experimentally establish the “structure–performance” relationships predicted by DFT, Ag-based metals were controllably loaded onto commercial TiO_2_ (anatase) nanoparticles. Guided by theoretical insights, we synthesized a series of samples and evaluated their POCM performance in a continuous-flow reactor under Xe-lamp irradiation. As shown in Figure 2a, bare TiO_2_ nanoparticles displayed negligible activity, yielding only CO_2_ at a rate of 1.2 mmol g^–1^ h^–1^ without producing any C_2_ products. In contrast, the introduction of Ag nanoparticles (TAg) significantly enhanced both activity and selectivity, achieving production rates of 6.46 mmol g^–1^ h^–1^ for C_2_H_6_ and 4.71 mmol g^–1^ h^–1^ for CO_2_, corresponding to a C_2_ selectivity of 72.3%. Incorporation of secondary metals further modulated performance. For example, Pt addition markedly promoted CO_2_ formation, while Au favored higher C_2_ yields. Most notably, TAgPd achieved outstanding performance, with production rates of 13.3 mmol g^–1^ h^–1^ (C_2_H_6_), 0.72 mmol g^–1^ h^–1^ (C_2_H_4_), 0.05 mmol g^–1^ h^–1^ (CO), and 2.43 mmol g^–1^ h^–1^ (CO_2_), corresponding to a remarkable C_2_ product selectivity of 92%. The different loading amounts were also scanned in Figure S5.

**Figure 2:**
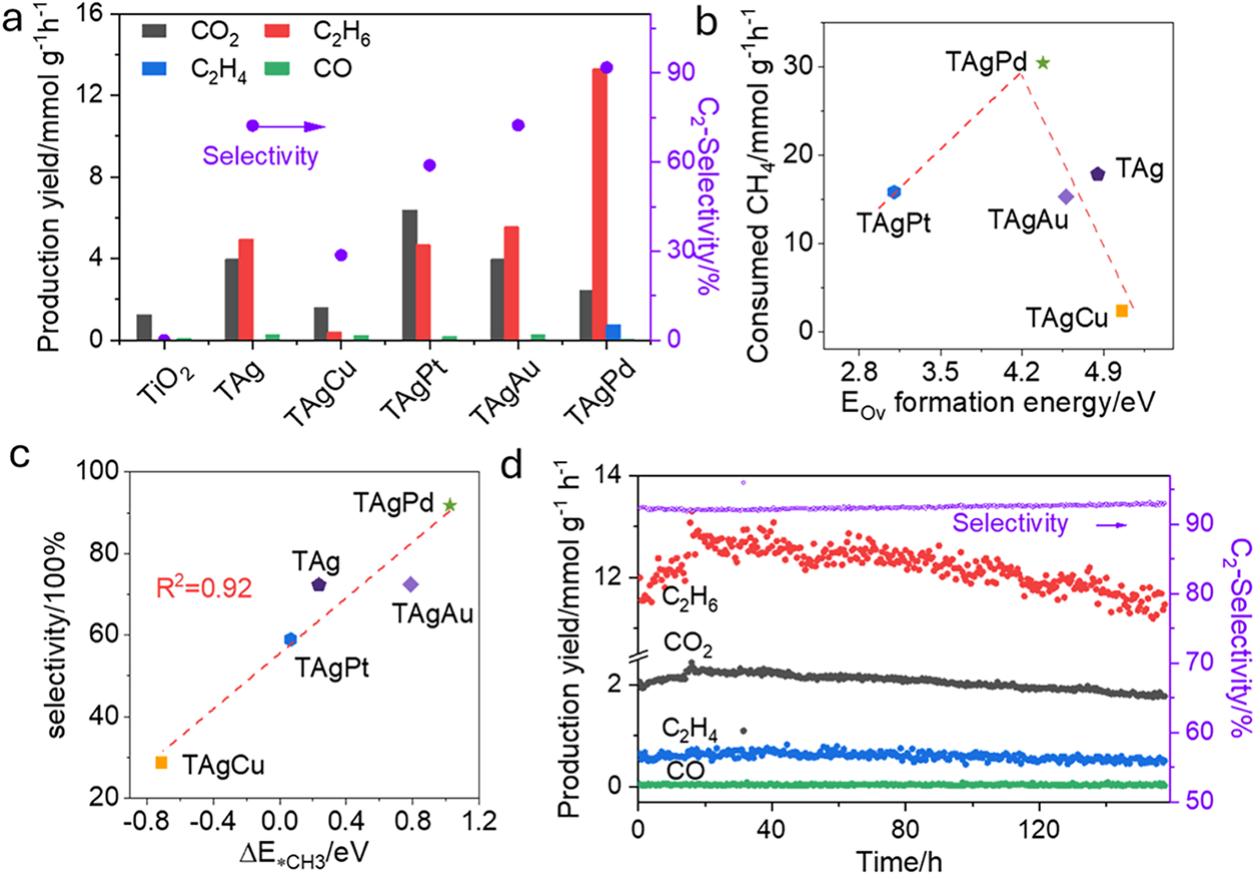
Experimental validation of activity-selectivity descriptors in POCM. (a) methane conversion rates and product distributions over a series of samples (including commercial TiO_2_, TAg, TAgCu, TAgPt. TAgAu and TAgPd) under light irradiation. The bar graph shows product yields for CO_2_ (black), C_2_H_6_ (red), C_2_H_4_ (blue), and CO (green), while the purple dots represent C_2_ selectivity (C_2_H_6_ + C_2_H_4_); (b) relationship between methane consumption and the calculated O_v_ formation energy for different metal cocatalysts supported on anatase TiO_2_, indicating that a proper O_v_ formation energy enhances methane activation; (c) selectivity toward C_2_ products (C_2_H_6_ and C_2_H_4_) as a function of ΔE_*CH_3__, showing that difference of CH_3_ adsorption energies favor C–C coupling over overoxidation; (d) stability test of the TAgPd catalyst over continuous operation. Catalyst loading: 5 mg deposited on glass fiber filter paper. Reaction conditions: CH_4_/O_2_ (125/1 mL·min^–1^) under illumination from a 300 W Xe lamp. The temporal evolution of CO_2_, C_2_H_6_, C_2_H_4_, and CO production, and C_2_ selectivity is plotted over time, demonstrating both high selectivity and good stability for C_2_ product formation.

To connect these experimental findings with the above theoretical calculation results, methane consumption rates were plotted against the calculated *E*_OV_ values in Figure 2b. A volcano-type relationship was observed, with TAgPd at the peak. On the right side of the volcano, higher *E*_OV_ values reflect less reactive lattice oxygen and reduced methane activation. On the left side, however, excessively low *E*_OV_ values (e.g., in TAgPt) did not translate to higher activity. Instead, the overly active lattice oxygen promotes extended dehydrogenation, driving methane to complete oxidation into CO_2_. Since the CH_4_ → CO_2_ pathway consumes more oxygen per molecule than the CH_4_ → C_2_H_6_ route, such systems exhibit slower overall methane conversion. In contrast, TAgPd provides a balanced lattice oxygen activity that enables efficient C–H activation while suppressing deep oxidation, consistent with the optimal catalytic performance observed.

Selectivity trends were then correlated with the ΔE_*CH_3__ descriptor, defined as the adsorption energy difference of *CH_3_ between TiO_2_ and metal sites ΔE_*CH_3__ (E_*CH_3_‑T_ – E_*CH_3_‑M_). As shown in Figure 2c, C_2_ selectivity increased linearly with ΔE_*CH_3__, highlighting that product distribution is governed by competitive CH_3_ adsorption. Systems where *CH_3_ preferentially binds to metal sites favor C–C coupling, while retention on the oxide surface leads to deep oxidation. TAgPd, with the largest ΔE_*CH_3__, exhibited the highest C_2_ selectivity, whereas TAgCu, with preferential adsorption on TiO_2_, displayed the lowest. The synergy of moderately low *E*_OV_ and large ΔE_*CH_3__ renders TAgPd the most effective system, delivering the highest C_2_H_6_ production rate and selectivity among all samples.

The stability of TAgPd was further assessed under continuous-flow operation. As shown in Figure 2d, no significant deactivation was observed around 160 h of reaction, with C_2_ product yields maintained around 14 mmol g^–1^ h^–1^ and selectivity consistently above 92%. The apparent quantum efficiency (AQE) for methane conversion reached 12.5% at 350 nm (Figure S6), placing TAgPd among the best-performing photocatalysts reported for POCM in terms of activity, selectivity, AQE, and long-term durability (Table S1 and Figure S7). Control experiments confirmed that the C_2_ products originate exclusively from methane conversion (Figure S8). No detectable hydrocarbons were observed when methane was replaced with Ar, or when photocatalyst or light irradiation was omitted. Postreaction characterizations further confirmed the robustness of TAgPd: synchrotron powder diffraction (PD, Figure S9) and X-ray photoelectron spectroscopy (XPS, Figure S10) revealed no structural changes, while TEM imaging (Figure S11) showed stable AgPd particle sizes. Raman spectroscopy (Figure S12) detected no coke formation. Together, these results verify the high durability of TAgPd under continuous gas–solid POCM conditions.

### Probing the Structure and M–MO Interaction of TAgPd

To further investigate the structure and interaction between metal and metal oxide, AgPd metal cocatalysts with tunable ratio of Ag and Pd were prepared. As shown in the Figure 3a, the as-synthesized metallic nanoparticles were loaded onto anatase with an average size of 4.5 nm (Figure S13) and displayed a lattice spacings of 0.20 nm attributed to (200) facet of Ag or Pd. Pristine commercial anatase TiO_2_ nanoparticles shows a lattice spacing of ∼0.36 nm, corresponding to the anatase (101) plane in Figure S14. After metal loading, the morphology and lattice structure of the TiO_2_ support remain essentially unchanged. In Figure S15, synchrotron-based powder diffraction (PD) patterns are collected with anatase TiO_2_ (JCPDS #21–1272) as the main peak and some small peaks attributed to the metallic nanoparticles. According to the typical (111) diffraction peak of Ag and Pd, we have observed a shift of the (111) peak position with increasing Pd contents in Figure S16. Vegard’s Law was applied to distinguish its structure from the single metal structure. As shown in Figure S17, it predicts a linear relationship between composition and lattice parameter, indicating the formation of a homogeneous solid solution. Furthermore, high-angle annular dark-field scanning transmission electron microscopy (HAADF-STEM) and corresponding energy dispersive X-ray (EDX) mapping were performed to further identify this nanostructure. The EDX mapping in Figure 3b reveals the homogeneous distribution of Ag and Pd, overlapping with the metallic crystal structure, confirming the mixture of the two metals, which further supports the PD analysis.

**Figure 3:**
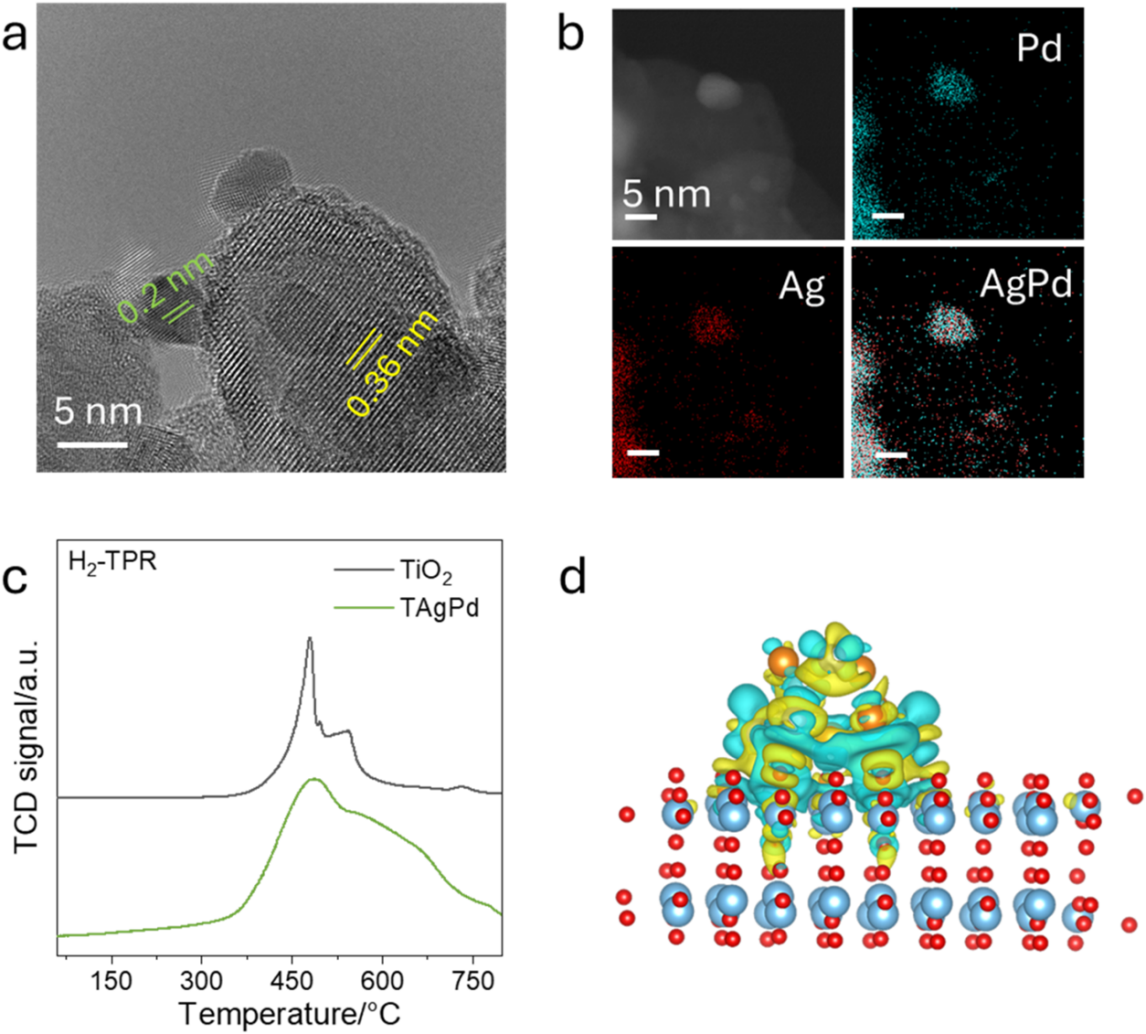
Structure of TAgPd and evidence of strong M–MO interaction. (a) High-resolution TEM (HRTEM) image illustrating clear lattice fringes of both AgPd particles and the anatase TiO_2_ support, with measured interplanar spacings; (b) HAADF-STEM image and corresponding EDX elemental maps of Ag, Pd, and the Ag–Pd overlap, confirming homogeneous distribution at the nanoscale. The scale bar is 5 nm; (c) hydrogen temperature-programmed reduction (H_2_-TPR) profiles collected under identical conditions for bare anatase (TiO_2_, gray) and TAgPd (green): TAgPd displays an earlier leading edge and a broader main reduction band with a long tail, indicating reduced activation barriers and a wider distribution of sites for lattice-oxygen removal; (d) charge different analysis of TAgPd shows the charge transfer between metal and anatase, indicating electrons transfer from metal to anatase. The iso-surface value is 0.001 e Å^–3^. Charge depletion and accumulation are labeled in cyan-color and yellow, respectively. Oxygen, titanium, sliver and palladium atoms are denoted as red, light blue, orange and dark blue balls, respectively.

To reveal the interaction effect of cocatalysts on the anatase, hydrogen temperature-programmed reduction (H_2_-TPR) was conducted, as shown in Figure 3c. Pristine anatase TiO_2_ exhibits a narrow reduction peak centered at 480 °C with a shoulder peak at 540 °C, attributed to the removal of surface and subsurface lattice oxygen. By contrast, TAgPd shows an earlier onset and a broadened main band with a long, gradual tail rather than the sharp peak and steep decay of anatase. This downshifted leading edge and extended tail signify a continuum of lowered activation barriers for oxygen release, consistent with strong electronic coupling at the AgPd-TiO_2_ interface that polarizes Ti–O bonds and facilitates vacancy formation and oxygen migration. Thus, the temperature distribution of the main peak and its tail demonstrates that lattice oxygen in TAgPd is intrinsically more labile and more reactive than in pristine anatase.

To probe the critical role of the M–MO interaction in governing interfacial charge redistribution, charge difference and Bader charge analysis were performed in Figure 3d. In the single-metal TAg system, Ag transfers a substantial 1.47 electrons directly to the oxide, reflecting a straightforward metal-to-oxide charge flow. In contrast, in the AgPd/TiO_2_ system, the redistribution becomes more complex: Ag donates 0.91 electrons while Pd accepts a fraction 0.29 electrons, and the net charge transfer to TiO_2_ is moderated (0.62 e^–^). This indicates that the introduction of a second metal adds an additional degree of freedom, where charge can be partitioned both between the metal components and across the metal-oxide interface. The charge redistribution was verified by the high-resolution X-ray photoelectron spectroscopy (XPS). In Figure S18, it indicates the Ag 3d peaks shift from 367.5 eV for pure Ag, negatively to 366.6 eV for AgPd. The 1.1 eV negative shift suggests that incorporating Pd increases the electron density in the Ag d-band while decreasing the electron density in the sp-band.^8^ This shift is consistent with previous reports.^8,29^ To probe the local atomic environment, X-ray absorption spectroscopy (XAS) of Ag and Pd K edge were conducted (Figure S19), respectively. Distance of Pd–Pd (Figure S20) almost keeps no change, while Figure S21 and Table S2 demonstrate that the distance of Ag–Ag decreased obviously compared to Ag foil, which is consistent with the observed lattice shrinkage.^8^

The above findings illustrate the crucial role of M–MO interactions in governing the performance. When metal nanoparticles are supported on the TiO_2_ surface, strong interfacial coupling arises that alters the electronic structure of both components. On the one hand, the deposited metals can donate or withdraw charges from the TiO_2_ lattice, thereby tuning the O 2p band onset and modulating the *E*_OV_. This directly affects the availability and reactivity of lattice oxygen species for C–H bond activation. On the other hand, the presence of TiO_2_ modifies the adsorption energetics of methyl intermediates on the metal sites, captured by the descriptor E_*CH3_. The balance of these two effects: oxygen vacancy formation on the oxide and intermediate stabilization on the metal, ultimately determines whether methane is efficiently activated and whether *CH_3_ couples selectively into C_2_ products or undergoes overoxidation. Thus, the M–MO interaction serves as the fundamental origin of the dual-descriptor framework, linking atomic-scale interfacial phenomena to catalytic activity and selectivity.

### Reaction Mechanisms investigations on TAgPd

To gain deeper insights into the POCM process on TAgPd, CH_4_ temperature-programmed desorption (CH_4_-TPD) was first conducted to evaluate methane adsorption on the catalyst surface. As shown in Figures 4a and S22, the introduction of metal significantly enhanced adsorption capacity, with desorption peaks shifting to lower temperatures and exhibiting larger areas compared to pristine TiO_2_ (531 °C). Notably, TAgPd displayed four distinct desorption peaks at 225, 336, 455, and 664 °C, indicative of multiple adsorption sites and enhanced methane activation ability relative to bare TiO_2_ and single-metal systems.

**Figure 4:**
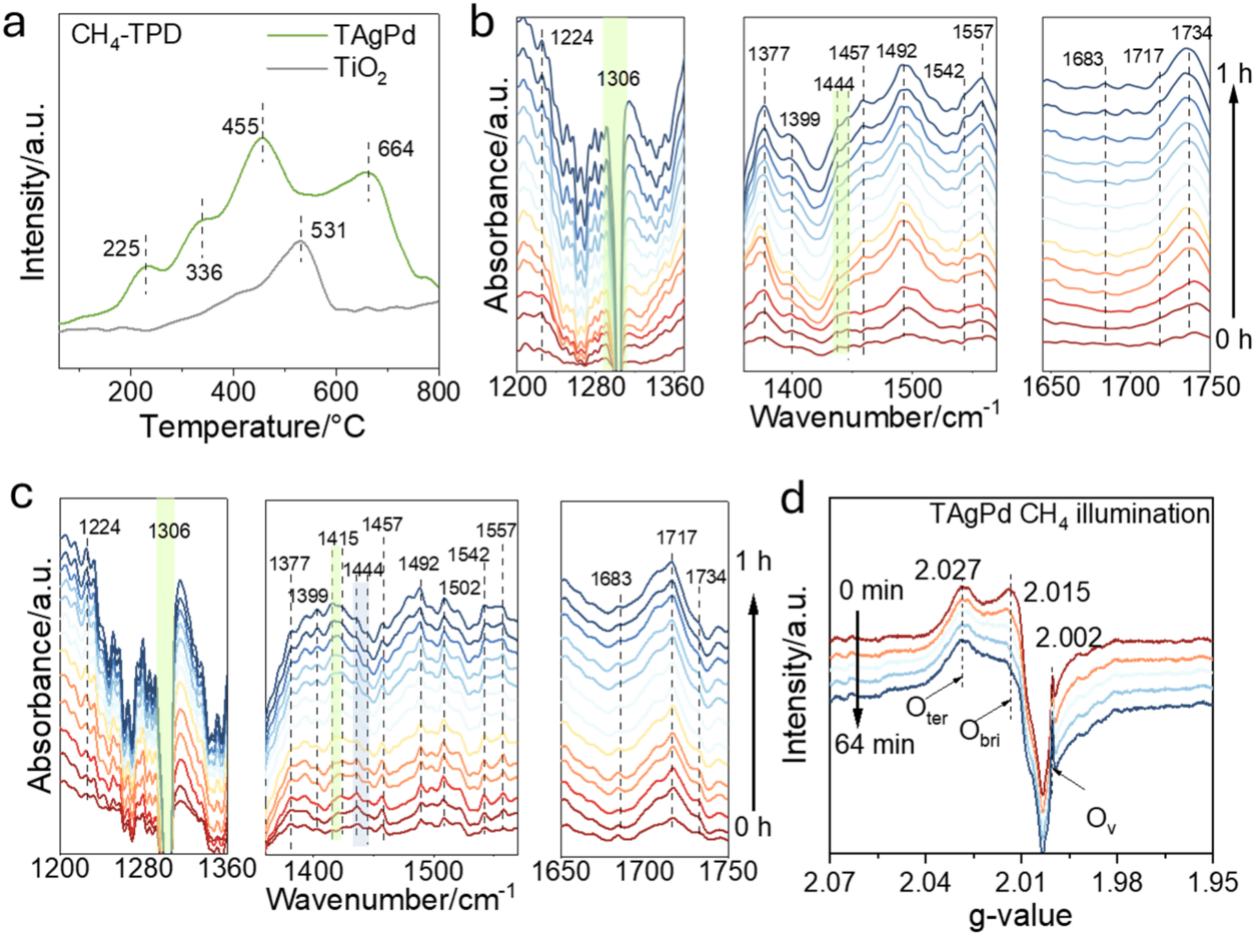
Mechanistic investigation of photocatalytic methane oxidation over various catalysts. (a) CH_4_-TPD spectrum of TAgPd and pristine TiO_2_; (b) in situ synchrotron-FTIR spectra under CH_4_/O_2_ (125/1 mL/min^–1^) flow over TAgPd catalyst with light irradiation from 0 to 60 min (red to blue), revealing the formation and evolution of surface intermediates including CH_X_, CH_3_O*, and HCOO*; (c) in situ synchrotron-FTIR spectra under CH_4_/O_2_ (125/1 mL min^–1^) over TAg catalyst; (d) Time-resolved in situ EPR spectra (0–64 min) tracking the dynamic evolution of the three species under photocatalytic methane activation.

To investigate methane activation pathways, in situ synchrotron-based Fourier-transform infrared (FTIR) microspectroscopy was performed for TAgPd (Figure 4b) and TAg (Figure 4c). Negative peaks observed within 1261–1355 cm^–1^ spectral range with the main peak at 1306 cm^–1^ indicated methane consumption under illumination. The first C–H bond cleavage led to *CH_3_ formation, observed at 1399 and 1492 cm^–1^ on both TAg and TAgPd.^30,31^ Subsequent oxidation yielded *OCH_3_ intermediates (1436, 1444 cm^–1^), precursors to CO_2_.^17,32^ Notably, TAg exhibited much stronger *OCH_3_ signals, suggesting their accumulation on Ag and their tendency to undergo further oxidation, consistent with higher CO_2_ selectivity. Indeed, downstream oxidation products including HCHO (1717, 1734 cm^–1^), HCOO^–^ (1415, 1423, 1542, 1557 cm^–1^), HCO_3_^–^ (1683 cm^–1^), and *CO_3_^2–^ (1224, 1397, 1502 cm^–1^) were observed predominantly on TAg,^17,20,31,33^ highlighting extensive overoxidation. By contrast, these intermediates appeared with much lower intensities on TAgPd, demonstrating that Pd incorporation suppresses overoxidation. Instead, new peaks at 1377 and 1457 cm^–1^, attributable to *CH_2_CH_3_ species, were detected on TAgPd but negligible on TAg, suggesting that Pd facilitates the C–C coupling and subsequent dehydrogenation of ethane to ethylene.^17^ Compared with the metal-loaded samples, bare TiO_2_ exhibits only very weak surface intermediate signals, indicating its limited ability to activate CH_4_ and generate detectable reaction intermediates under photocatalytic conditions (Figure S23).

To probe reactive surface species under working conditions, in situ electron paramagnetic resonance (EPR) spectroscopy was employed. Under N_2_, TAgPd exhibited an intrinsic oxygen vacancy signal at *g* = 2.00249, which intensified under illumination (Figure S24), confirming light-induced vacancy formation.^34^ Upon O_2_ exposure, the signal intensity decreased, consistent with vacancy refilling. In contrast, pure anatase (Figure S25) showed signals at *g* = 1.985 (Ti^3+^), indicating photogenerated electrons trapped by Ti^4+^.^34^ The absence of Ti^3+^ signals in TAgPd suggests that electrons are effectively extracted from TiO_2_ by the alloy nanoparticles, preventing bulk charge trapping. When methane was introduced, new oxygen-related species were observed (Figure 4d). Signals at *g* = 2.015, 2.011, and 2.001 were assigned to bridging O^•–^ (O_bri_) species, while those at *g* = 2.027, 2.013, and 2.004 correspond to terminal O^•–^ (O_ter_) species formed via hole trapping at lattice oxygen sites.^15,34^ The progressive increase of the *g* = 2.002 signal indicated the creation of oxygen vacancies during methane activation. Importantly, the O_ter_ signal remained stable throughout the 0–64 min illumination period, suggesting hole accumulation in lattice oxygen. The simultaneous buildup of O_bri_ and only limited vacancy formation indicates that lattice oxygen extraction is kinetically difficult, making vacancy formation a likely rate-determining step, consistent with prior reports.^19^ Upon introduction of mixed CH_4_/O_2_ feeds (Figure S26), both O_ter_ and O_bri_ signals diminished, confirming their active participation in the catalytic cycle. In the absence of gaseous O_2_, that is, under a pure CH_4_ feed, TAgPd exhibits a pronounced decline in catalytic performance relative to that observed under mixed CH_4_/O_2_ conditions (Figure S27). Only very limited product formation is observed, indicating that O_2_ plays a crucial role in sustaining methane conversion in this system. This result suggests that O_2_ is not merely a background component but is essential for maintaining the active oxygen species and enabling efficient catalytic turnover. Therefore, O_2_ is a key participant in the reaction network and is indispensable for achieving high activity under the present POCM conditions.

Based on the combined in situ EPR and synchrotron-FTIR results, lattice oxygen activation and vacancy formation are central to methane activation, but the selectivity of subsequent steps depends critically on how *CH_3_ intermediates are stabilized at the M–MO interface. In TAgPd, efficient electron extraction prevents Ti^3+^ accumulation, balanced lattice oxygen activity enables controlled C–H activation, and the interfacial environment suppresses OCH_3_ overoxidation while promoting C–C coupling pathways. This mechanistic evidence provides direct spectroscopic validation of the DFT-predicted descriptors (*E*_OV_ and ΔE_*CH_3__), establishing a coherent picture of why TAgPd achieves both high activity and selectivity in POCM.

### Materials Design Principles

To elucidate the combined effects of *E*_OV_ and ΔE_*CH_3__ on methane activation and C_2_ product formation, we carried out a two-dimensional correlation analysis (Figure 5). As shown in Figure 5a, methane conversion rate and C_2_ selectivity are positively correlated: catalysts that activate methane more efficiently also favor multicarbon products over undesired byproducts. This observation underscores that efficient methane activation intrinsically facilitates C–C coupling. The mechanistic origin of this trend can be traced to the dual descriptors. Methane conversion is primarily governed by the reactivity of lattice oxygen, reflected in *E*_OV_. Lower

**Figure 5:**
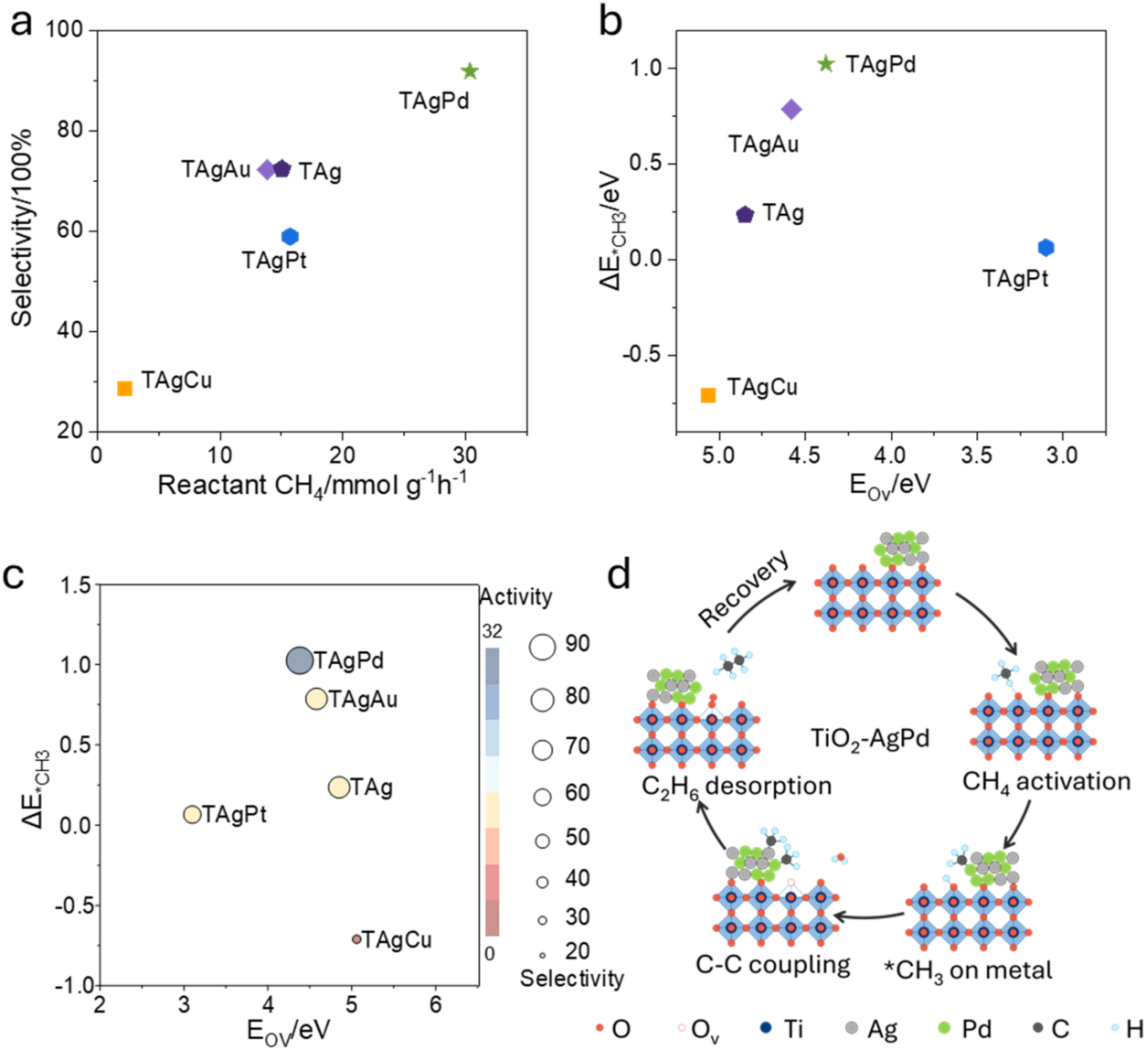
Correlation between catalyst descriptors, methane conversion, and product selectivity. (a) Relationship between methane conversion rate and C_2_ (C_2_H_4_ + C_2_H_6_) selectivity, showing a positive correlation where enhanced methane activation favors C–C coupling. (b) Two-dimensional volcano-like trend between oxygen vacancy formation energy (*E*_OV_) and methyl adsorption energy difference (ΔE_*CH_3__), highlighting the synergistic influence of lattice oxygen reactivity and methyl adsorption energetics on catalytic performance. The deviation observed for TAgPt indicates that excessively low *E*_OV_ may lead to overoxidation, reducing C_2_ selectivity despite high methane conversion. (c) Two-dimensional descriptor map correlating catalytic performance with oxygen-vacancy formation energy (*E*_OV_) and methyl adsorption-energy difference (ΔE_*CH_3__). The symbol color denotes catalytic activity (consumed methane/mmol g^–1^ h^–1^), and the symbol size represents selectivity (C_2_ products/%). The distribution of the data points highlights the coupled influence of lattice-oxygen reactivity and methyl adsorption energetics on overall performance. (d) Schematic illustration of the proposed photocatalytic reaction loop on AgPd/TiO_2_.

*E*_OV_ values correspond to more labile oxygen reservoirs, enabling facile C–H bond cleavage and higher conversion rates. In contrast, C_2_ selectivity is dictated by ΔE_*CH_3__, which determines the relative adsorption preference of methyl intermediates between oxide and metal sites, thereby controlling whether *CH_3_ radicals undergo coupling or deep oxidation. The interplay of these two factors is further illustrated in Figure 5b, which reveals a volcano-type dependence of performance, which shows the similar relationship in Figure 2b. On the ascending branch, moderately low *E*_OV_ values promote methane activation, while larger ΔE_*CH_3__ differences favor selective CH_3_ coupling, delivering both high conversion and selectivity. However, when *E*_OV_ becomes excessively low, oxygen species become overly reactive, driving CH_3_ toward full oxidation into CO_*x*_ rather than C–C coupling. For example, TAgPt lies on this descending side of the volcano, where ultralow *E*_OV_ suppresses C_2_ selectivity despite facile C–H activation. By contrast, TAgPd resides in the optimal window, achieving a balance between lattice oxygen reactivity and methyl adsorption energetics that maximizes both methane conversion and C_2_ selectivity. These findings establish a general design principle for POCM: co-optimization of lattice oxygen activity (*E*_OV_) and methyl adsorption balance (ΔE_*CH_3__) is essential to achieve high performance. More broadly, this dual-descriptor framework provides predictive guidance for the design of advanced photocatalysts in selective oxidation and C–C coupling reactions, where strong bond activation must be delicately balanced against the suppression of overoxidation pathways. To deepen our understanding, the two-dimensional map was shown in Figure 5c. It correlates catalytic performance with *E*_OV_ and ΔE_*CH_3__. Here, the symbol color represents activity, and the symbol size reflects selectivity. The map shows that high activity is associated with a relatively large ΔE_*CH_3__, whereas high selectivity appears in the region combining a large *E*_OV_ with a small ΔE_*CH_3__. Among the examined samples, TAgPd is in the high-activity and the highest selectivity region. Figure 5d summarizes the proposed photocatalytic reaction loop on AgPd/TiO_2_. Under illumination, methane is activated at the metal–semiconductor interface to form surface methyl species, accompanied by H transfer and the involvement of lattice oxygen from TiO_2_. The generated methyl intermediates subsequently undergo coupling to produce C_2_ hydrocarbons, while the oxide surface is regenerated through the interfacial redox cycle. This scheme highlights the cooperative roles of the AgPd bimetallic sites and the TiO_2_ support, in which the metal component promotes methane activation and intermediate coupling, whereas the oxide lattice participates in oxygen transfer and catalyst turnover.

To examine the generality of this framework, AgPd was further supported on different oxide hosts, including Anatase-O_V_, ZnO, CeO_2_, and commercial TiO_2_ (P25). As shown in Figure S28, these systems exhibited markedly different activities and C_2_ selectivity, indicating that M–MO interactions modulate both the metal and oxide components and thereby reshape the overall catalytic behavior. Accordingly, the correlation among *E*_OV_, ΔE_*CH_3__ and catalytic performance is not universal in a single quantitative sense but depends on the specific interfacial interaction. This generality is not limited to oxide variation alone: the cocatalyst identity is also an integral part of the M–MO interaction, and different metals are likewise expected to alter electronic coupling, oxygen reactivity, and methyl-site preference, leading to distinct yet related *E*_OV_, ΔE_*CH_3__ landscapes. Thus, while the dual-descriptor framework captures the essential trade-off governing POCM, each metal/oxide family may define its own descriptor map. This result highlights an important direction for the field: developing catalyst-family specific descriptor landscapes that integrate both metal and oxide contributions, enabling a more predictive design strategy for selective methane conversion.

## Conclusion

Through a combined theoretical-experimental investigation, we established a dual-descriptor framework that rationalizes and predicts activity and selectivity of POCM. Density functional theory calculations identified two interfacial parameters *E*_OV_ and ΔE_*CH_3__ as decisive factors controlling activity and selectivity. *E*_OV_ reflects the availability and reactivity of lattice oxygen, with moderately low values promoting efficient C–H activation, whereas excessively low values drive CH_3_ overoxidation into CO_*x*_. In parallel, ΔE_*CH_3__ governs the stabilization and fate of CH_3_ intermediates, where balanced adsorption energetics enable migration to metal sites and selective C–C coupling into C_2_ hydrocarbons, while unbalanced adsorption favors complete oxidation. Ag-based systems supported on TiO_2_ validate this framework: AgPd achieves the optimal balance of descriptors, delivering a C_2_ yield of 14 mmol g^–1^ h^–1^ with 92% selectivity, an AQE of 12.5% at 350 nm, and stability around 160 h. In situ EPR and synchrotron-FTIR results further confirm that vacancy dynamics and moderated CH_3_ stabilization at the metal–oxide interface underpin the superior selectivity of AgPd. Extending this analysis to different oxide supports reveals that each host defines its own *E*_OV_–ΔE_*CH_3__ landscape, highlighting the support-dependent nature of descriptor–performance relationships. Collectively, these results demonstrate that *E*_OV_ and ΔE_*CH_3__ enable a quantitative description of M–MO interactions by linking interfacial electronic structure to catalytic function, thereby providing a generalizable design principle for selective methane conversion and a predictive framework for understanding metal–metal oxide interfaces.

## Supplementary Material



## Notes

Detailed experimental procedures, catalyst synthesis details, material characterization (PD, XPS, TEM, XAS, FTIR, EPR, CH_4_-TPD), photocatalytic performance measurements, DFT computational results (PDF)
