# Supplementary material for: Metal–Metal Oxide Interaction Modulated Photocatalytic Methane Conversion
Source: J Am Chem Soc. 2026 Apr 16;148(16):17094–103. doi: 10.1021/jacs.6c01783 (PMC13133787; doi:10.1021/jacs.6c01783)
Supplement: Supplementary file 1 [file ja6c01783_si_001.pdf]

Metal−Metal Oxide Interaction Modulated Photocatalytic Methane Conversion

Yanzhao Zhang[1], Jiakang You[1], Kai Wang[1,2], Dazhi Yao[3], Haijiao Lu[1], Jitraporn Vongsvivut[4], Jingwei Hou[1], Peng Chen[1], Yonggang Jin[3], Gang Liu[5,6], Zhiliang Wang[1], Lianzhou Wang[1,2*]*

1. Nanomaterials Centre, School of Chemical Engineering and Australian Institute for Bioengineering and Nanotechnology, The University of Queensland, St Lucia, QLD 4072, Australia.
   Email: zhiliang.wang@uq.edu.au, lianzhou.wang@polyu.edu.hk
2. Department of Applied Biology and Chemical Technology, The Hong Kong Polytechnic University, Hong Kong SAR, China
3. Commonwealth Scientific and Industrial Research Organization (CSIRO) Mineral Resources, 1 Technology Court, Pullenvale, QLD, 4069, Australia.
4. Infrared Microspectroscopy (IRM) Beamline, ANSTO-Australian Synchrotron, Clayton, VIC 3168, Australia.
5. Shenyang National Laboratory for Materials Science, Institute of Metal Research, Chinese Academy of Sciences, 72 Wenhua Road, Shenyang, 110016, China.
6. School of Materials Science and Engineering, University of Science and Technology of China, 72 Wenhua Road, Shenyang, 110016, China.

Methods

Chemicals: Titanium dioxide (TiO_2 anatase, nanopowder, < 25 nm particle size), titanium dioxide (TiO_2 P25, nanopowder), cerium (IV) oxide (CeO_2, nanopowder, < 25 nm particle size), silver nitrate ($AgNO_3$), gold (III) chloride trihydrate ($HAuCl_4 \cdot 3H_2O$), palladium (II) chloride ($PdCl_2$), copper (II) chloride ($CuCl_2$), chloroplatinic acid hexahydrate ($H_2PtCl_6 \cdot 6H_2O$), and sodium borohydride ($NaBH_4$) were purchased from Sigma-Aldrich. Zinc oxide (ZnO, nanoparticles < 30 nm) was obtained from Maikun Chemical. All the chemicals are analytical reagents and used directly without any further purification. Ultrapure water was used throughout all experiments.

Synthesis of metal nanoparticles on anatase TiO_2. Metal cocatalysts (monometallic Ag/Pd, binary metallic AgPd, AgPt, AgAu and AgCu) were loaded on the metal oxides by a simple $NaBH_4$ reduction method. In the typical synthesis, 0.2 g commercial anatase nanoparticles were dispersed in 10 mL deionized water by ultrasonication for 30 min. Subsequently, a certain amount of 10 mM $AgNO_3$ aqueous solution (2.8 mL) and 10 mM $PdCl_2$ (2.8 mL) were added, followed by stirring for 15 min. Then 5 mL $NaBH_4$ solution (1 mg/mL) was dropped into the above-mixed solution. After stirring for 3 hours, the products were collected and washed with water three times and dried at 60 °C for 12 h. Other cocatalysts were synthesized by the similar methods with the same loading amount as sample AgPd.

Different AgPd ratios samples were prepared by adding different amounts of $AgNO_3$ (4.9, 4.2, 2.8, 1.4 mL) and $PdCl_2$ solution (0.7, 1.4, 2.8, 4.2 mL).

Synthesis of AgPd on different metal oxides. Different metal oxides as listed in the above chemicals section were loaded with the same amount of Ag and Pd as AgPd via the same methods above. Anatase with oxygen vacancy denoted as Ana-O_v was prepared by the $NaBH_4$ reduction methods. The same weight of commercial anatase powder and $NaBH_4$ powder were ground in a mortar for 10 min. The mixture was calcined in Argon at 350 °C for 30 min. The resulting powder was washed with water three times and dried at 60 °C for 12 h. The Ana-O_V was used as the substrate material for loading AgPd.

Materials Characterization. Raman spectroscopy was carried out using a confocal Raman microscope (RM5, Edinburgh Instruments) with a 10× objective and a 532 nm laser in all experiments. The time-resolved photoluminescence (TRPL) decay studies were carried out with a 377 nm pulsed diode laser excitation source on a fluorescence spectrophotometer (FLSP-900, Edinburgh Instruments). High-resolution transmission electron microscopy (HRTEM)

images and energy-dispersive X-ray spectroscopy (EDS) were obtained on the FEI Titan Thermis TEM (200 kV) with scanning TEM (STEM) mode. Valance band XPS spectra measurement was performed on a VGESCALAB 210 XPS spectrometer with Mg Kα source. The binding energies were referenced to the C 1s peak at 284.8 eV. UV-visible diffuse reflectance spectra were obtained on a UV-Vis spectrophotometer (UV2600, Shimadzu, Japan). The synchrotron-based X-ray absorption, powder diffraction, and FTIR were obtained from the XAS, PD and IRM beamlines at the Australian Synchrotron (Clayton, Victoria), respectively. The XAS raw data was background-subtracted, normalized and Fourier-transformed using Athena software.

Photocatalytic activity tests. The photocatalytic performance tests were conducted in 50 mL flow reactor made of stainless steel (PLR-GPTR 50, Beijing Perfectlight Technology Co., Ltd.) with a quartz window at ambient conditions. A 300 W Xenon lamp source (full spectrum, PLS-SXE300+, Beijing Perfectlight Technology Co., Ltd.) was employed as the light source. In a typical test, 5 mg of photocatalysts were dispersed in water then loaded on the glass fibre filter (Whatman) and dried. Before illuminating, the reactor was purged with CH_4 and O_2 (125/1 min^{-1}) as the feeding gas at a total flow rate of 126 mL min^{-1} for 20 min to exclude air. The feeding gas keeps flowing during illumination. The as-obtained gaseous POCM product was analysed by the gas chromatograph (GC-2014, Shimadzu), via online injection from the reactor 30 min per sample testing. The batch mode experiments were conducted in the same environment with no gas flowing. Blank/control experiments were conducted under the corresponding conditions, namely, photocatalysts in ultra-high purity Argon (>99.999%) under illumination, photocatalysts in ultra-high purity CH_4 without illumination, and ultra-high purity CH_4 under illumination without photocatalysts.

The C_2 product selectivity was calculated based on the conservation of carbon (Equation 1):

$$C_2 \text{ selectivity} = (2(n(C_2H_6) + n(C_2H_4))) / (2(n(C_2H_6) + n(C_2H_4)) + n(CO_2) + n(CO)) \times 100\% \qquad \text{(Equation 1)}$$

where n (product) represents the moles of the specific products.

The apparent quantum efficiency (AQE) was calculated according to the following equation (Equation 2):

$$AQE = N(\text{electrons})/N(\text{photons})*100\% \qquad \text{(Equation 2)}$$

where N(electrons) and N(photons) represent the number of reacted electrons and the number of incident photons, respectively.

According to the chemical equations ($2CH_4+1/2O_2 \rightarrow C_2H_6+H_2O$; $4CH_4+O_2 \rightarrow C_2H_4+2H_2O$; $8CH_4+3O_2 \rightarrow CO_2+4H_2O$), the reacted electrons for each product can be calculated according to the following equation (Equation 3):

$$N(\text{electrons}) = 2\times N(C_2H_6) + 4\times N(C_2H_4) + 8\times N(CO_2)$$

$$= 2\times n(C_2H_6)\, N_A + 4\times n(C_2H_4)\, N_A + 8\times n(CO_2)\, N_A \quad \text{(Equation 3)}$$

where N(product) and N_A represent the molecular number for the specific product and Avogadro's constant (6.02×10^{23} mol $^{-1}$), respectively.

The number of incident photons was calculated according to the following equation (Equation 4):

$$\text{N(photons)} = I \times A \times \lambda / (h \times c \times \text{EQE(Si)}) \quad \text{(Equation 4)}$$

where I is the light intensity measured by a photodetector (W cm^{-2}), A is the irradiation area, λ is the wavelength of the incident light, h is Planck constant, c is the speed of light, EQE(Si) is the external quantum efficiency of the detector at certain wavelength.

***In situ* FTIR Microspectroscopy.** The *in situ* synchrotron-FTIR measurements were carried out on the Infrared Microspectroscopy (IRM) beamline at the Australian Synchrotron (Clayton, Victoria). The experiment was performed in reflectance mode using a homemade gas-tight photocatalytic cell with a 0.50-mm-thick CaF_2 top window. The IRM beamline was equipped with a Bruker Vertex 80v spectrometer coupled with a Hyperion 3000 FTIR microscope and a liquid nitrogen-cooled narrow-band mercury cadmium telluride (MCT) detector (Bruker Optik GmbH, Ettlingen, Germany). All the synchrotron-FTIR spectra were recorded with a 15× objective (N.A. = 0.40) within a spectral range of 3800−700 cm^{-1} using 256 co-added scans and 4-cm^{-1} spectral resolution. Blackman-Harris 3-Term apodization, Mertz phase correction, and zero-filling factor of 2 were set as default acquisition parameters using OPUS 8 software suite (Bruker Optik GmbH, Ettlingen, Germany). The background spectrum was collected after purging reactant gas until it was stable. After that, a series of sample spectra were recorded under irradiation conditions as a function of time to investigate the dynamics of surface reaction.

***In situ* electron paramagnetic resonance (EPR) tests.** *In situ* EPR spectra were collected on a Bruker Elexsys E500 spectrometer equipped with an ElexSys Super High Sensitivity

Probehead and liquid nitrogen cooling using a cryogen-free cryostat (Bruker waveguide Cryogen-free system with recirculatory, WVGD SYS 5K F70H wRCRC 2). The magnetic field was calibrated with a Gauss meter, and measurements were conducted using a modulation amplitude of 0.8 mT, a modulation frequency of 100 kHz and a microwave power of 5 mW (10 dB of 200 mW, non-saturating condition). A quartz tube was used for the reactor. 0.1g catalysts were put in the quartz tube and purged with nitrogen for the first test. Illumination was on. Spectrum was collected after different gases were purged.

Computational Methods. All DFT calculations were performed with the Vienna Ab Initio Simulation Package (VASP) code.[1] The Perdew-Burke-Ernzerhof (PBE) functional was employed for electron exchange-correlation within the generalized gradient approximation.[2] The projector-augmented wave (PAW) method was used to describe the ionic cores.[3] The geometry optimizations were performed with a 520 eV cut-off energy for plane wave expansion. The ionic relaxations were conducted until all the forces were smaller than 0.02 eV·$Å^{-1}$. A Gaussian smearing was used with 0.2 eV width and a (2 × 2 × 1) Gamma k-point grid was applied. The Tkachenko-Scheffler method was employed to describe long-range van der Waals interactions.[4] The TiO_2 (anatase) was optimized in a 2 × 2 × 1 supercell. Ag based nanoparticle models were built based on the optimized anatase model, respectively. The whole anatase model were 128 titanium atoms, 64 oxygen atoms, 5 silver atoms and 5 gold, platinum, palladium and copper atoms respectively according to the different models. All the structures were relaxed first and conducted for the intermediates, charge transfer and oxygen vacancy formation energy calculations. A vacuum space of 15 Å was applied to separate the interactions between neighboring slabs. For the bimetallic clusters, different initial atomic arrangements of the two metals were considered without imposing a specific ordered distribution. After full structural relaxation, the lowest-energy optimized configuration was selected for subsequent calculations. Oxygen-vacancy formation is site-dependent, several top-surface oxygen atoms at different positions were examined for each model. Oxygen atoms far from the metal/oxide interface gave E_{ov} values close to those of pristine TiO_2, whereas interfacial oxygen atoms were consistently more labile. Accordingly, the reported E_{ov} values were taken from the top-surface oxygen site in the M–MO interfacial region.

The oxygen vacancy formation energy (E_{ov}) on the metal oxide slab was calculated according to: $E_{ov} = E_{def} + ½E_{O2} – E_{perf}$ where E_{def}, E_{perf} and E_{O2} are the total energies of the defective slab containing one oxygen vacancy, the corresponding pristine slab, and an isolated oxygen

molecule, respectively. This definition corresponds to the removal of one lattice oxygen atom from the slab under O-rich conditions, with the removed oxygen referenced to half of an oxygen molecule. All structures were fully relaxed before the energy evaluation.

The methyl adsorption energy (E_{CH3-M}) on the metal sites of the slab was calculated according to: $E_{CH3-M} = E_{*CH3} – E_{M} – E_{CH3}$, where E_{*CH3}, E_{M} and E_{CH3} are the total energies of the slab with adsorbed methyl, the clean slab, and the isolated CH_3 radical, respectively. The methyl adsorption energy (E_{CH3-MO}) on the metal oxides of the slab was calculated according to the similar quotation: $E_{CH3-MO} = E_{*CH3} – E_{MO} – E_{CH3}$.

E_{ov} and ΔE_{*CH3} (ΔE_{*CH3}, = $E_{*CH3-M} − E_{*CH3-MO}$) were derived from ground-state DFT calculations and are used here as descriptors of the intrinsic interfacial chemistry rather than explicit excited-state observables. In this framework, E_{ov} reflects lattice-oxygen lability, while ΔE_{*CH3} describes the relative thermodynamic preference of methyl species for different interfacial sites.

The O 2p position discussed in this work is defined as the onset edge of the occupied O 2p states in the projected density of states (PDOS).

Supplementary Figures

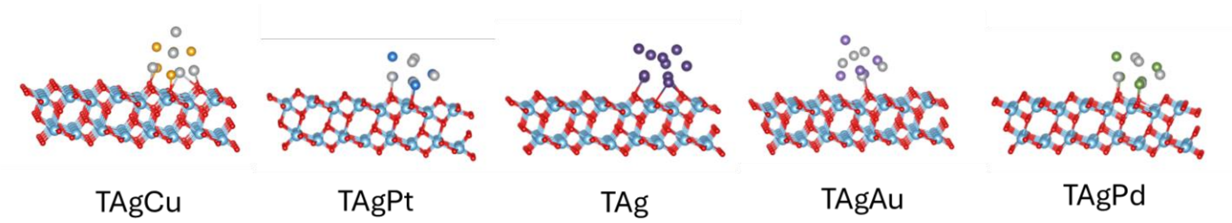

Figure S1. DFT-optimized structures of the mono- and bimetallic cluster/TiO_2 models used in this study, including TAgCu, TAgPt, TAg, TAgAu, and TAgPd. These snapshots illustrate the optimized geometries of the supported metal clusters on the anatase TiO_2 surface employed in the DFT calculations.

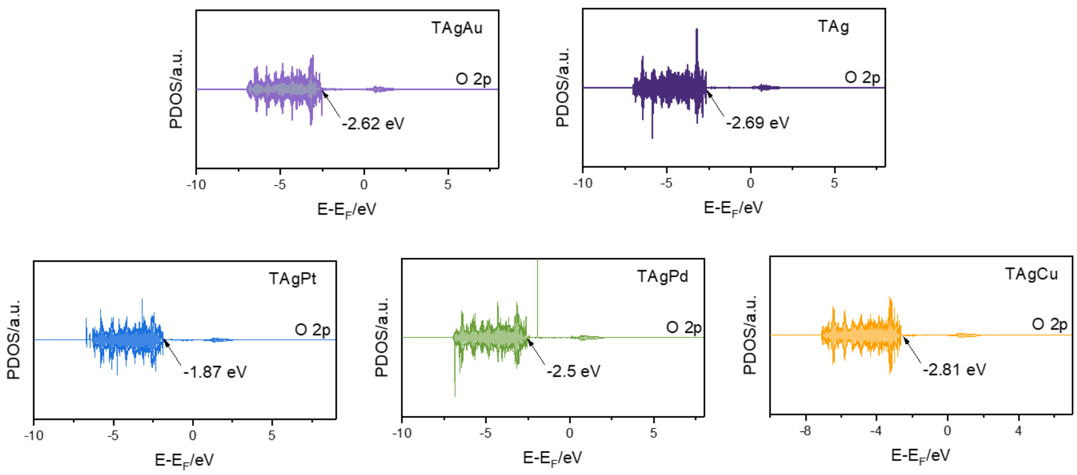

Figure S2. O 2p projected density of states of TAg, TAgAu, TAgPt, TAgPd and TAgCu.

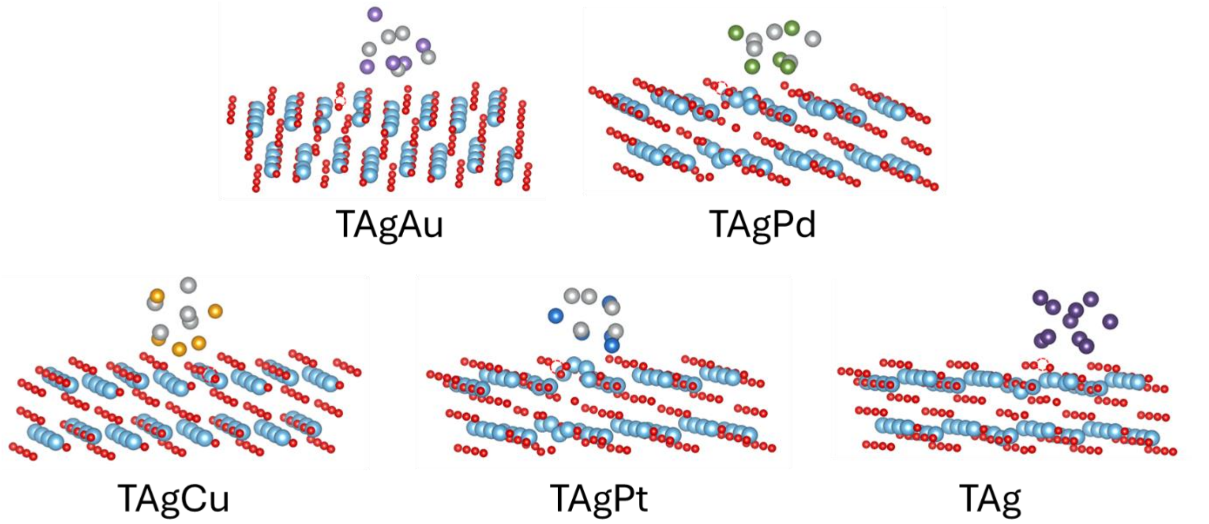

Figure S3. DFT-optimized structures of oxygen-vacancy models for the investigated M–MO/TiO_2 systems. The oxygen vacancy is outline as dash circle. In each case, the vacancy was created by removing a top-surface lattice oxygen atom in the metal–oxide interfacial region, followed by full structural relaxation. These optimized configurations illustrate the local structural response of TiO_2 and the supported metal clusters to interfacial oxygen removal.

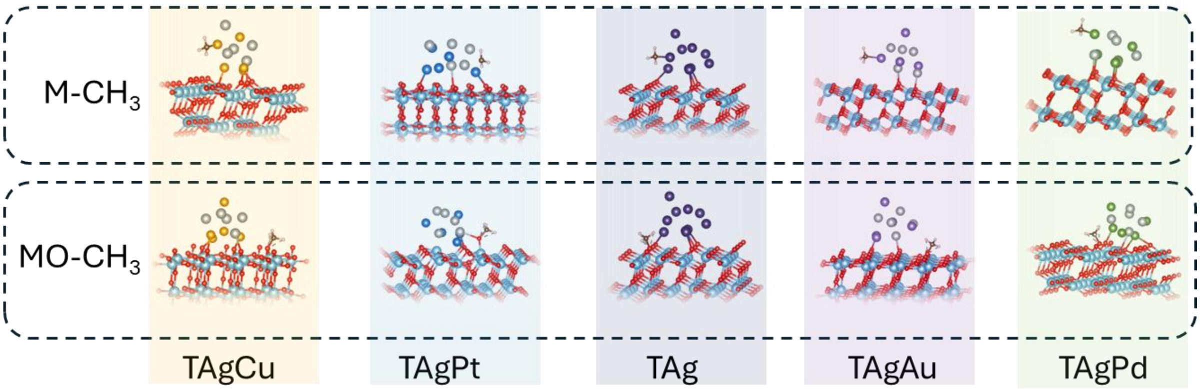

Figure S4. DFT-optimized adsorption configurations considered on the Ag-based mono- and bimetallic/TiO_2 models.

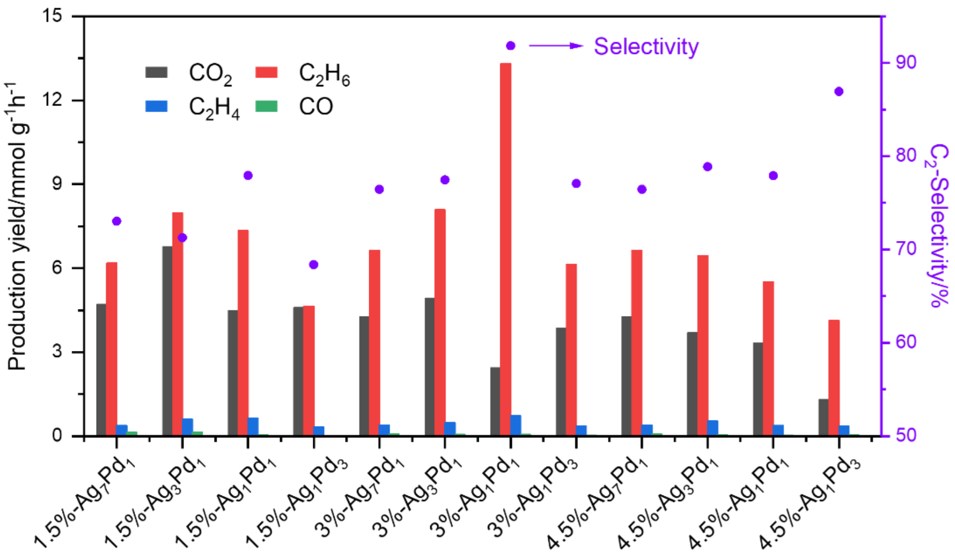

Figure S5. Composition-dependent photocatalytic performance of AgPd/TiO_2 catalysts. Product formation rates over AgPd/TiO_2 with different total metal loadings (1.5, 3.0, and 4.5 wt%) and Ag/Pd atomic ratios (7:1, 3:1, 1:1, and 1:3) under identical reaction conditions. Gray, red, blue, and green bars represent the formation rates of the detected products, and the purple symbols denote C_2 selectivity.

To examine the effect of catalyst composition, a series of AgPd/TiO_2 samples with different total metal loadings (1.5, 3.0, and 4.5 wt%) and Ag/Pd atomic ratios (7:1, 3:1, 1:1, and 1:3) were evaluated (Figure S5). The catalytic performance strongly depends on both parameters. Among all samples, 3%-Ag1Pd1/TiO_2 shows the highest C_2 hydrocarbon productivity and C_2 selectivity, indicating the most favorable balance between methane activation and C–C coupling. These trends suggest that Ag mainly contributes to methane activation, whereas Pd tunes the local electronic and adsorption properties of the bimetallic sites, thereby influencing intermediate stabilization and product distribution. Meanwhile, the total metal loading controls the density of metal–TiO_2 interfacial sites, with an intermediate loading being optimal for maximizing performance.

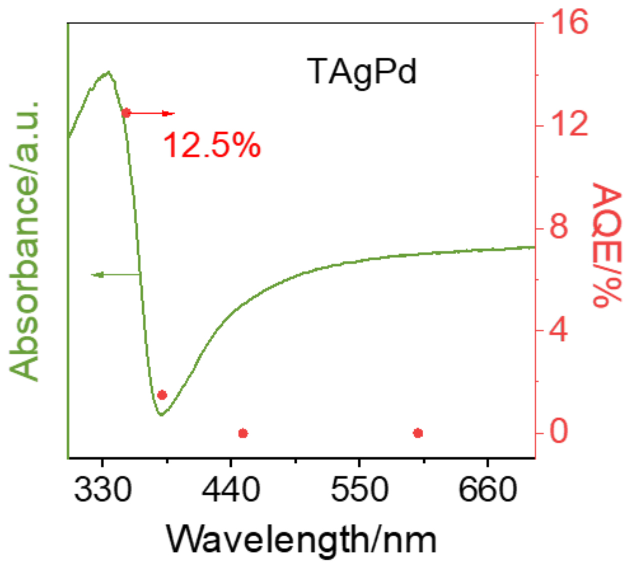

Figure S6. UV-visible spectrum and wavelength dependence of AQE for POCM on TAgPd. (AQE tested under the monochromatic light filter of 350, 380, 450 and 600 nm)

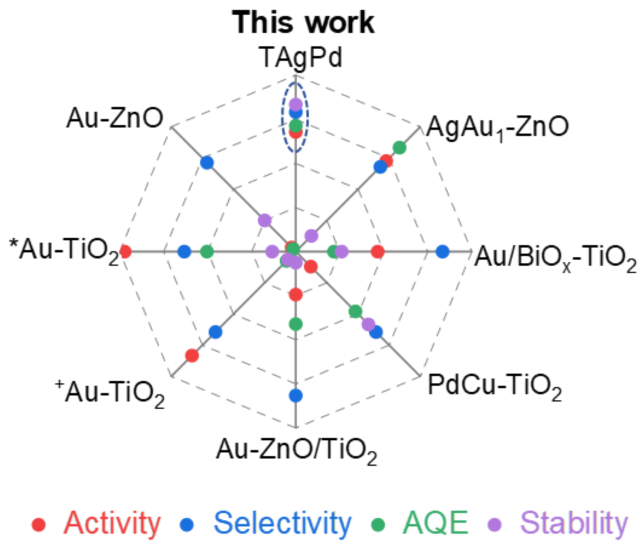

Figure S7. Radar chart summarizing POCM activity, selectivity, AQE and stability of different samples based on the reported references. The same reports are listed in the Table S1.

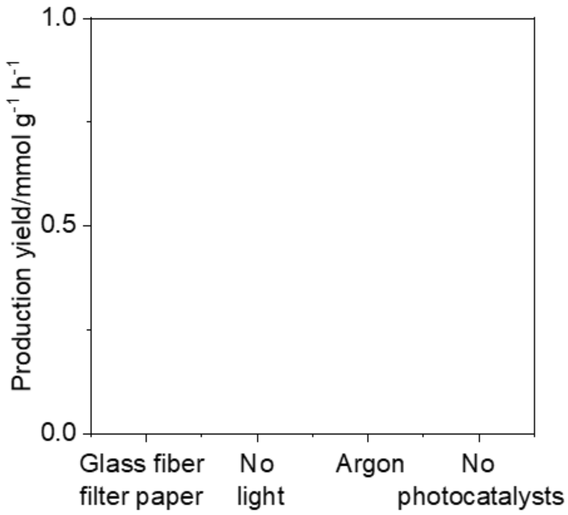

Figure S8. Blank experiments conducted in different conditions: glass fiber filter paper under illumination; in dark; Ar replace feeding gas; no catalysts in the reactor. All these blank experiments show no products indicating the products in performance tests are from methane conversion.

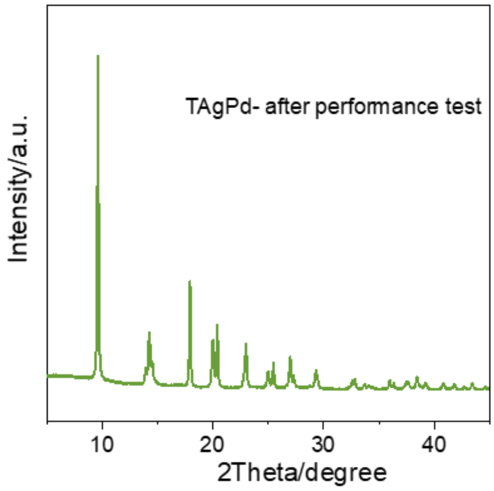

Figure S9. Synchrotron-based powder diffraction of TAgPd after performance test.

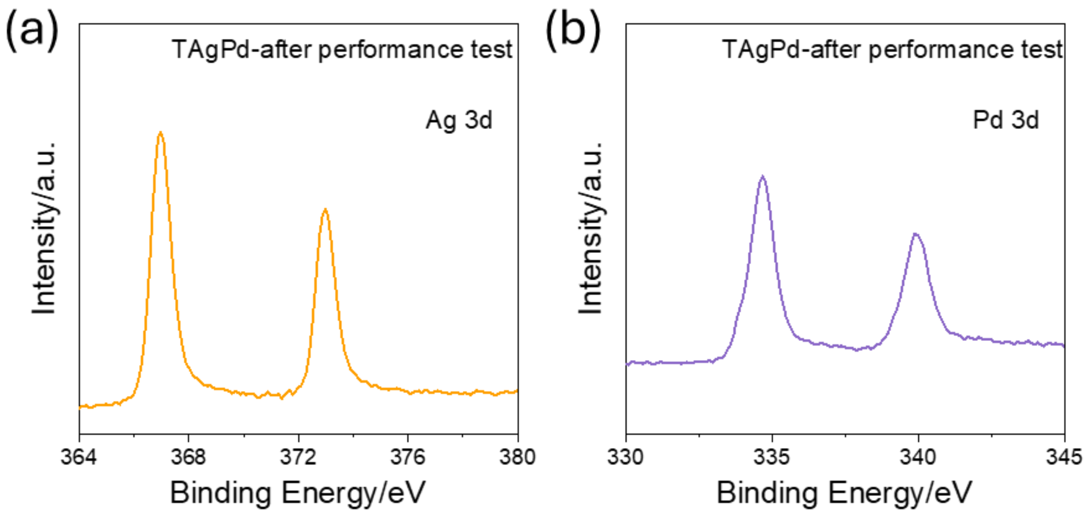

Figure S10. (a) Ag 3d and (b) Pd 3d XPS spectra of TAgPd after POCM performance test.

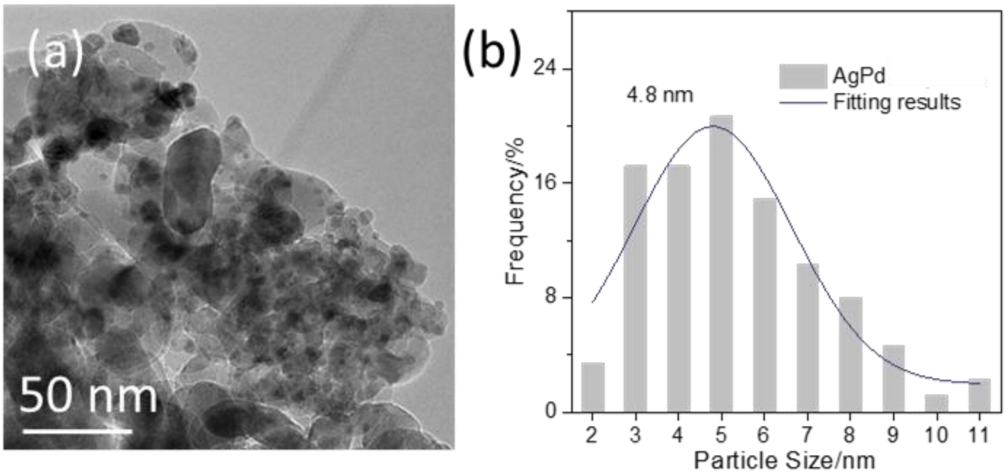

Figure S11. (a) TEM image and (b) corresponding AgPd particle size distribution of TAgPd after POCM performance testing.

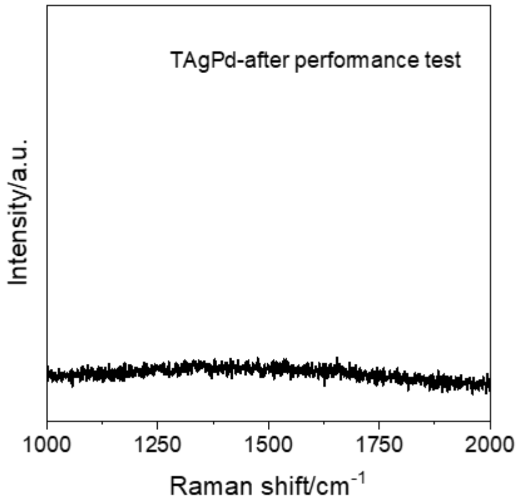

Figure S12. Raman spectrum of TAgPd after POCM performance test.

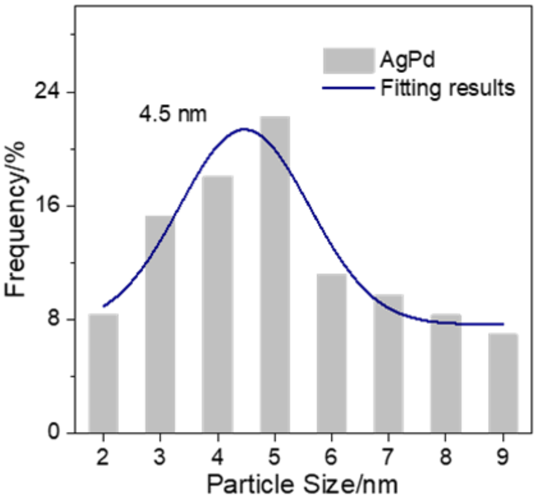

Figure S13. Size distribution of AgPd nanoparticles in the TAgPd sample. After fitting the different size portions, the average size for nanoalloy is around 4.5 nm.

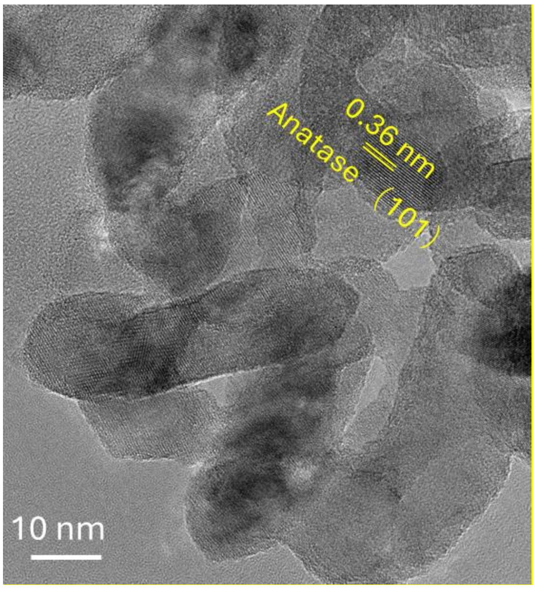

Figure S14. TEM image of commercial pristine TiO_2 anatase nanoparticles.

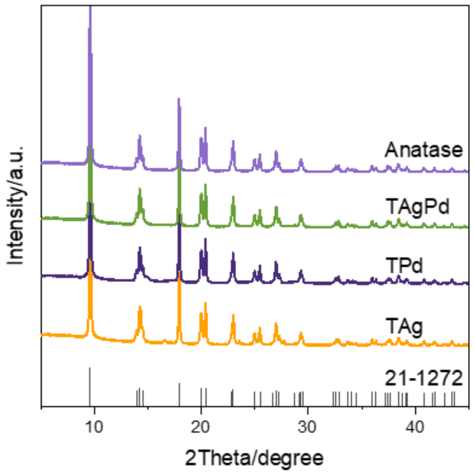

Figure S15. Synchrotron-based powder diffraction of TAg, TPd, TAgPd and pure anatase samples. The main peaks are from anatase, attributed to anatase (JCPDS 21-1272), with small peaks from Ag, Pd or AgPd nanoparticles.

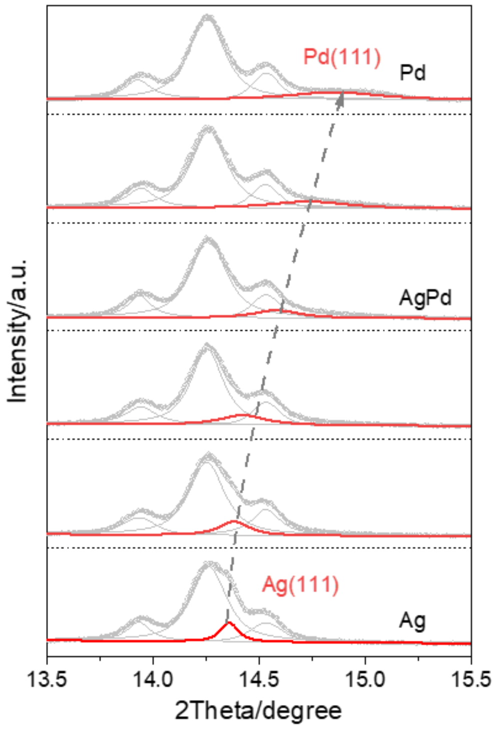

Figure S16. Synchrotron-based PD patterns of different Ag/Pd ratios (Pd contents increasing from bottom to up lines) loaded anatase highlighting the (111) peaks (red lines) of nanometal particles and peaks (grey lines) attributed to anatase with nanometals.

The (111) peak from nanoalloy diffraction overlapped with anatase peaks within the range of 13.5° to 15.5°, as indicated by the split red peak in Figure S11. monometallic Ag or Pd nanoparticle loaded anatase, denoted as Ag and Pd, exhibited peaks at 14.35° and 14.88°, respectively, corresponding to the (111) facets of the face-centred cubic (fcc) crystal structure. Similarly, bimetallic Ag-Pd nanoparticles at all compositions exhibited fcc structures, with the (111) peak ranging between the Ag (111) and Pd (111) peaks, suggesting the formation of an Ag-Pd alloy. The centre of the (111) peak shifted to higher angles with increasing Pd composition.

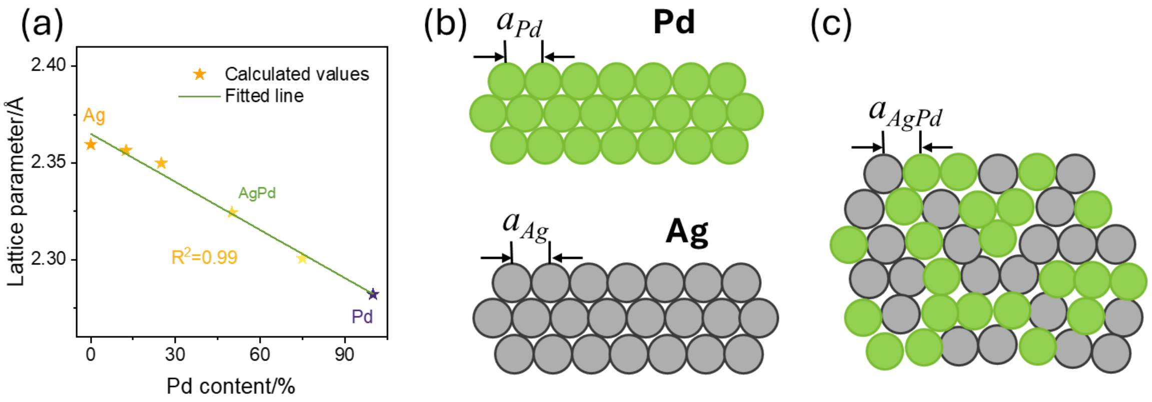

Figrue S17. (a) Vegard's Law plot showing the linear correlation between the (111) diffraction peak position and the nominal Pd content, confirming successful formation of AgPd solid solutions; (b, c) Schematic representations of atomic arrangements for monometallic Pd and Ag particles (b) and AgPd alloy particles (c), with corresponding lattice parameters (a_{Pd}, a_{Ag} and a_{AgPd}). Ag and Pd atoms are denoted as grey and green spheres, respectively.

Vegard's Law predicts that the lattice parameter (a_{AgPd}) varies linearly with the atomic fractions of Ag (a_{Ag}) and Pd (a_{Pd}), as illustrated schematically in Figure S12 b and S12 c. Pure Ag and Pd adopt fcc structures with distinct lattice constants, while the AgPd alloy shows a mixed atomic arrangement (x: the concentration of Pd in AgPd) and an intermediate lattice parameter:

$$a_{AgPd}=x^* \, a_{Pd}+(1-x)^* a_{Ag}$$

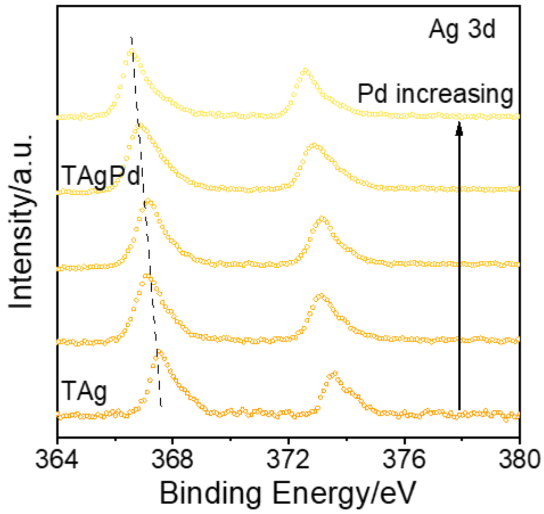

Figure S18. Ag 3d XPS spectra of different Ag compositions (Pd contents increasing from bottom to up lines) for metal-loaded anatase.

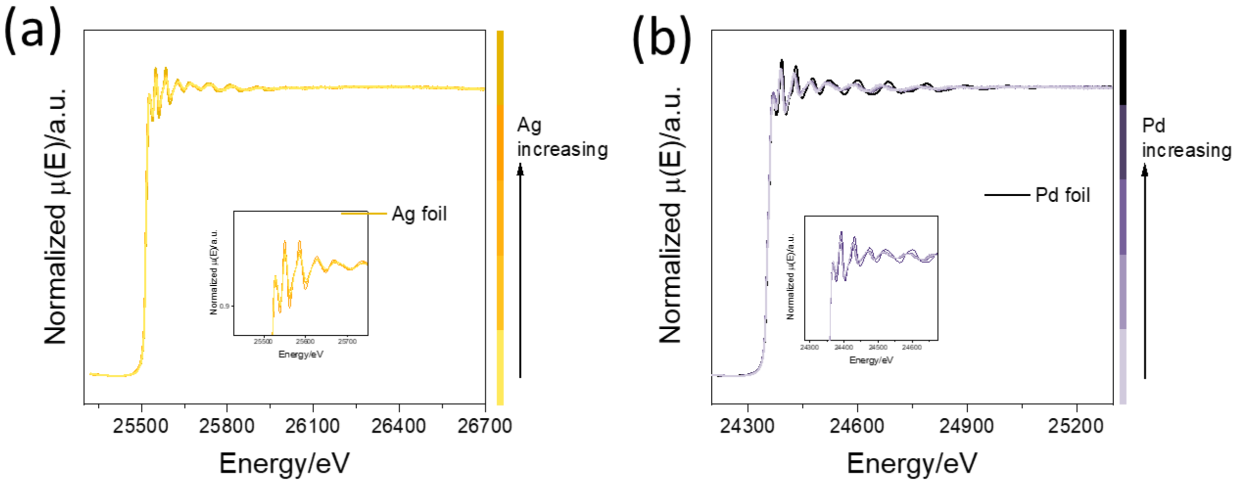

Figure S19. XAS spectra of (a) Ag K edge and (b) Pd K edge for different Ag/Pd ratios on anatase. (Pd contents increasing from dark to light color lines).

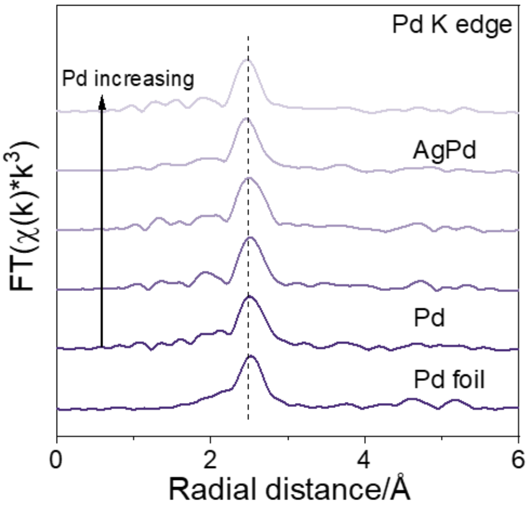

Figure S20. Pd K-edge FT-EXAFS of different Ag/Pd ratios on anatase. (Pd contents increasing from bottom to up lines).

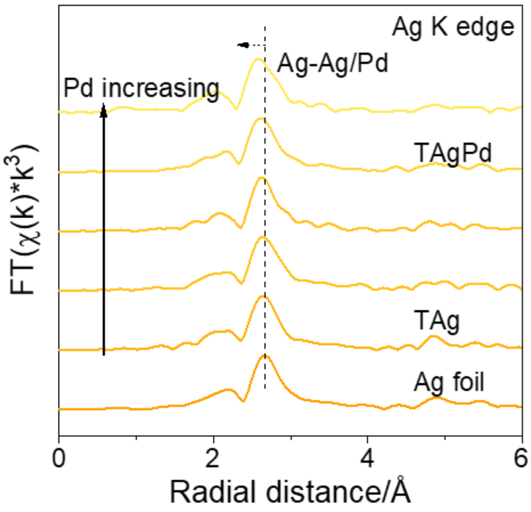

Figure S21. Ag K-edge FT-EXAFS of different Ag/Pd ratios on anatase (Pd contents increasing from bottom to up lines).

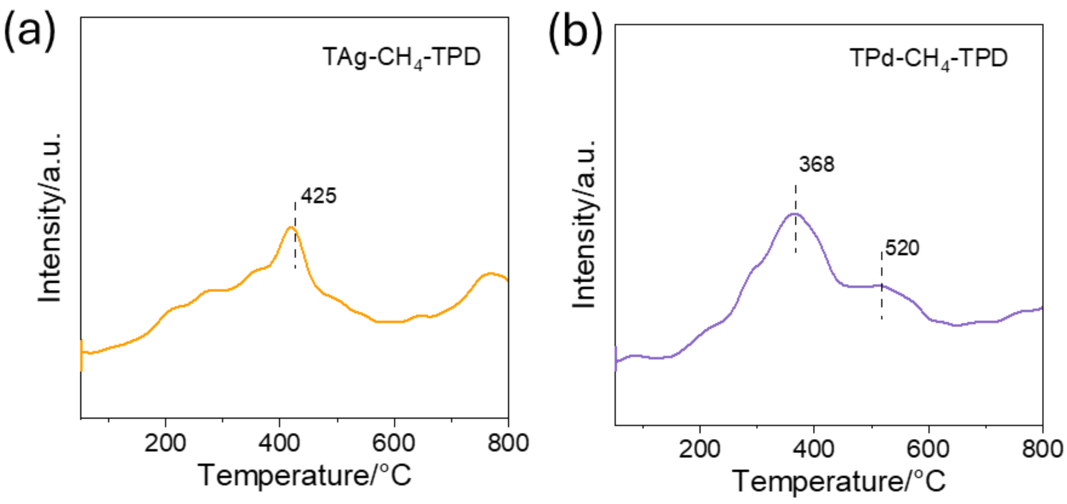

Figure S22. CH_4-TPD spectra of (a) TAg and (b) TPd.

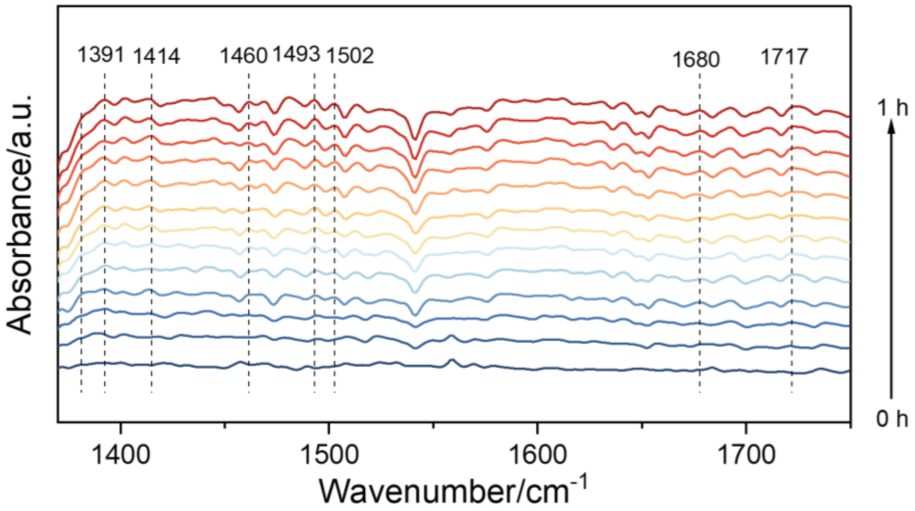

Figure S23. In situ FTIR spectra of pristine anatase TiO_2 collected under POCM reaction conditions as a function of irradiation/reaction time from 0 to 1 h.

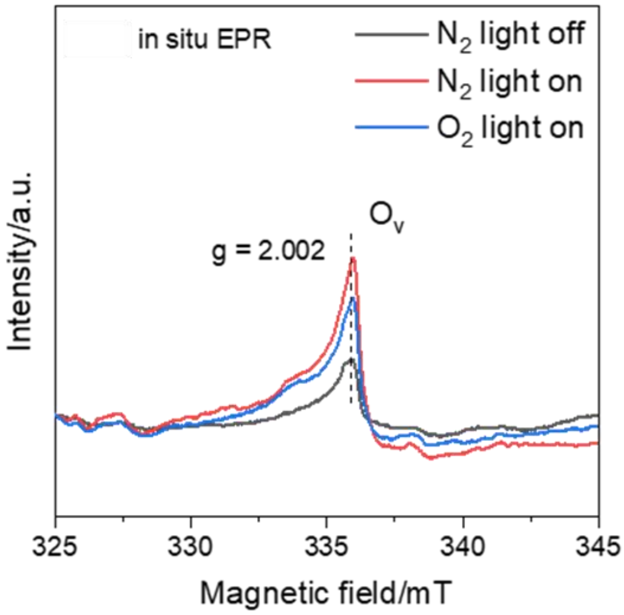

Figure S24. *In situ* EPR test for TAgPd sample with different illumination and gas conditions.

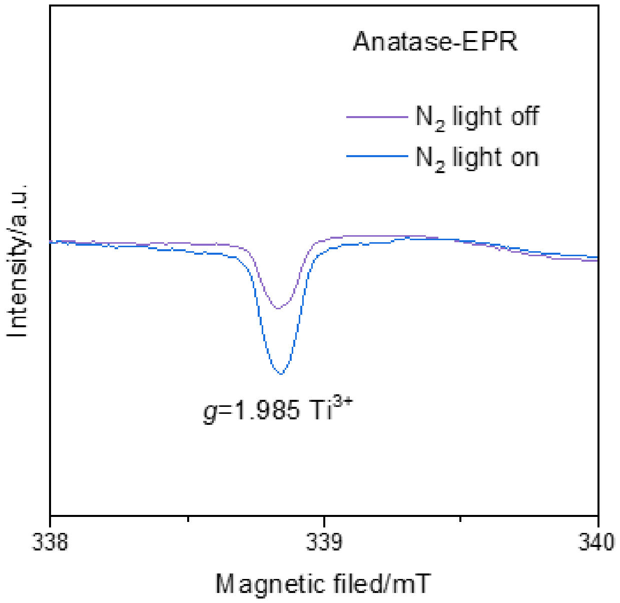

Figure S25. EPR test for pure anatase sample in N_2 with light on and off.

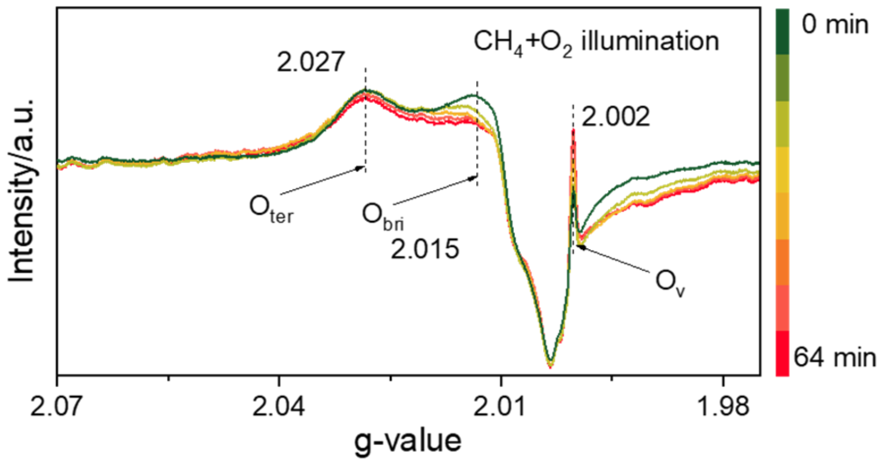

Figure S26. *In situ* EPR test for TAgPd sample from 0 min to 64 min.

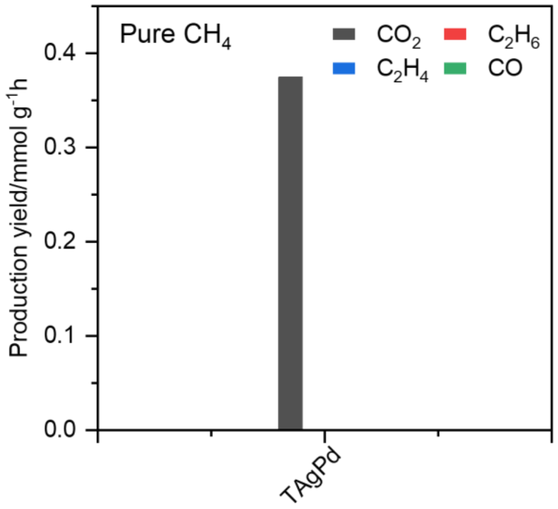

Figure S27. Catalytic performance of the TAgPd sample under pure CH_4 feed.

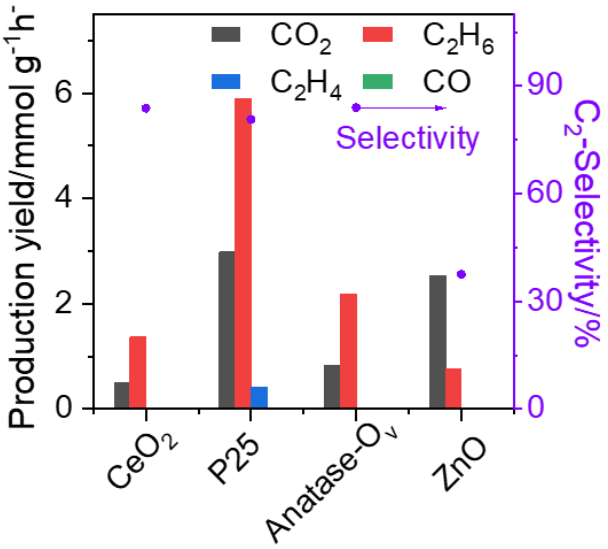

Figure S28. Photocatalytic oxidative coupling of methane (POCM) performance and C2 products selectivity of AgPd on the different metal oxides including CeO_2, P25, Ana-O_v (Anatase with oxygen vacancy) and ZnO.

Table S1. POCM performance comparison with the reported materials under similar conditions

Catalysts	Reaction conditions			Performance			Ref.
	Feeding gas	Light	AQE/ %	C_{2+} products/ mmol g^{-1} h^{-1}	C_{2+} selectivity/%	Stability/ h	
Anatase-AgPd	$CH_4 + O_2$ (125 + 1 mL/min)	300 W Xenon lamp	12.5 % at 350 nm	14	92	160	This work
ZnO-Au	$CH_4 + O_2$ (99 + 1)	365 nm LED light	0.38 % at 365 nm	0.683	83	48	5
ZnO-Au_1Ag_9	CH_4 + air (49.5 + 0.5)	300 W Xenon lamp	14.6 % at 350 nm	14	79	24	6
ZnO/TiO_2-Au	CH_4 + air (69 + 1)	300 W Xenon lamp	7.2 % at 360 nm	5.02	90	12	7
Au-TiO_2	$CH_4 + O_2$ (440 + 1); multipoint reactor	300 W Xenon lamp	1.3 % at 320-780 nm	18.7	90	240	8
Au-TiO_2	CH_4 + air (320 + 12); 65 °C	365 nm LED light;	10.3 % at 350 nm	15.3	90	30	9
Au-TiO_2/BiO_x	$CH_4 + O_2$ (70 + 1)	300 W Xenon lamp	3.8 % at 365 nm	9.6	97	50	10
PdCu-TiO_2	$CH_4 + O_2$ (373 + 1)	365 nm LED light	8.4 % at 365 nm	2.48	75	112	11

Table S2. Fitted results for Ag K-edge EXAFS for AgPd

Sample	Path	N/ Coordination Number	R/Å	ΔE_0/eV	σ^2/Å^2	R-factor
AgPd	Ag–Ag/Pd	8.2	2.83	0.142	0.00988	0.017

R = the length of Ag–Ag or Ag–Pd path, N = coordination number of metal atoms corresponding to Ag–Ag or Ag–Pd path, σ^2 = Debye-Waller factor, and ΔE_0 = inner potential correction.

References

[1] Kresse, G.; Furthmuller, Efficient iterative schemes for ab initio total-energy calculations using a plane-wave basis set. *J. Phys. Rev. B: Condens. Matter Mater. Phys*., **1996**, 54, 11169-11186.

[2] Perdew, J.P.; Burke, K.; Ernzerhof, M. Generalized gradient approximation made simple *Phys. Rev. Lett.,* **1996**, 77, 3865-3868.

[3] Kresse, G.; Joubert, D. From ultrasoft pseudopotentials to the projector augmented-wave method. *Phys. Rev. B: Condens. Matter Mater. Phys.,* **1999**, 59, 1758-1775.

[4] Tkatchenko, A.; Scheffler, M. Accurate molecular Van Der Waals interactions from ground-state electron density and free-atom reference data. *Phys. Rev. Lett.,* **2009**, 102, 073005.

[5] Wang, P.; Shi, R.; Zhao, Y.; Li, Z.; Zhao, J.; Zhao, J.; Waterhouse, G. I. N.; Wu, L.-Z.; Zhang, T. Selective photocatalytic oxidative coupling of methane via regulating methyl intermediates over Metal/ZnO Nanoparticles. *Angew. Chem., Int. Ed.* **2023**, *62* (23), e202304301. DOI: https://doi.org/10.1002/anie.202304301.

[6] Wang, Y.; Hong, G.; Zhang, Y.; Liu, Y.; Cen, W.; Wang, L.; Wu, Z. Photocatalytic oxidative coupling of methane over Au_1Ag single-atom alloy Modified ZnO with oxygen and water vapor: Synergy of gold and silver. *Angew. Chem., Int. Ed.* **2023**, *62* (42), e202310525.

[7] Song, S.; Song, H.; Li, L.; Wang, S.; Chu, W.; Peng, K.; Meng, X.; Wang, Q.; Deng, B.; Liu, Q.; et al. A selective Au-ZnO/TiO_2 hybrid photocatalyst for oxidative coupling of methane to ethane with dioxygen. *Nat. Catal.* **2021**, *4* (12), 1032-1042. DOI: 10.1038/s41929-021-00708-9.

[8] Chen, Y.; Zhao, Y.; Liu, D.; Wang, G.; Jiang, W.; Liu, S.; Zhang, W.; Li, Y.; Ma, Z.; Shao, T.; et al. Continuous flow system for highly efficient and durable photocatalytic oxidative coupling of methane. *J. Am. Chem. Soc.* **2024**, *146* (4), 2465-2473. DOI: 10.1021/jacs.3c10069.

[9] Li, X.; Li, C.; Xu, Y.; Liu, Q.; Bahri, M.; Zhang, L.; Browning, N. D.; Cowan, A. J.; Tang, J. Efficient hole abstraction for highly selective oxidative coupling of methane by Au-sputtered TiO_2 photocatalysts. *Nat. Energy* **2023**, *8* (9), 1013-1022. DOI: 10.1038/s41560-023-01317-5.

[10] Zhai, G.; Cai, L.; Ma, J.; Chen, Y.; Liu, Z.; Si, S.; Duan, D.; Sang, S.; Li, J.; Wang, X.; et al. Highly efficient, selective, and stable photocatalytic methane coupling to ethane enabled by lattice oxygen looping. *Sci. Adv. 10* (26), eado4390. DOI: 10.1126/sciadv.ado4390.

[11] Li, X.; Wang, C.; Yang, J.; Xu, Y.; Yang, Y.; Yu, J.; Delgado, J. J.; Martsinovich, N.; Sun, X.; Zheng, X.-S.; et al. PdCu nanoalloy decorated photocatalysts for efficient and selective

oxidative coupling of methane in flow reactors. *Nat. Commun.* **2023**, *14* (1), 6343. DOI: 10.1038/s41467-023-41996-y.
